# Population structure determines the tradeoff between fixation probability and fixation time

**DOI:** 10.1038/s42003-019-0373-y

**Published:** 2019-04-23

**Authors:** Josef Tkadlec, Andreas Pavlogiannis, Krishnendu Chatterjee, Martin A. Nowak

**Affiliations:** 1 IST Austria, A-3400 Klosterneuburg, Austria; 2 Lab for Automated Reasoning and Analysis, EPFL, CH-1015 Lausanne, Switzerland; 3 Program for Evolutionary Dynamics, Department of Organismic and Evolutionary Biology, Department of Mathematics, Harvard University, Cambridge, MA 02138 USA

**Keywords:** Evolutionary theory, Evolution

## Abstract

The rate of biological evolution depends on the fixation probability and on the fixation time of new mutants. Intensive research has focused on identifying population structures that augment the fixation probability of advantageous mutants. But these amplifiers of natural selection typically increase fixation time. Here we study population structures that achieve a tradeoff between fixation probability and time. First, we show that no amplifiers can have an asymptotically lower absorption time than the well-mixed population. Then we design population structures that substantially augment the fixation probability with just a minor increase in fixation time. Finally, we show that those structures enable higher effective rate of evolution than the well-mixed population provided that the rate of generating advantageous mutants is relatively low. Our work sheds light on how population structure affects the rate of evolution. Moreover, our structures could be useful for lab-based, medical, or industrial applications of evolutionary optimization.

## Abstract (web summary)

Josef Tkadlec et al. investigate population structures that mediate the trade-off between fixation probability and time. They identify structures that enable higher effective rates of evolution than the well-mixed population, which could be applied to in vitro evolution applications.

## Introduction

The two primary forces that drive evolutionary processes are mutation and selection. Mutation generates new variants in a population. Selection chooses among them depending on the reproductive rates of individuals. Evolutionary processes are intrinsically random. A new mutant that is initially present in the population at low frequency can go extinct due to random drift. The key quantities of evolutionary dynamics which affect the rate of evolution are^1–5^ (a) the mutation rate *μ*, which is the rate at which new mutants are generated; (b) the fixation probability *ρ*, which is the probability that the lineage of a mutant takes over the whole population; and (c) the fixation time *τ*, which is the expected time until the lineage of a mutant fixates in the population.

**Fig. 1:**
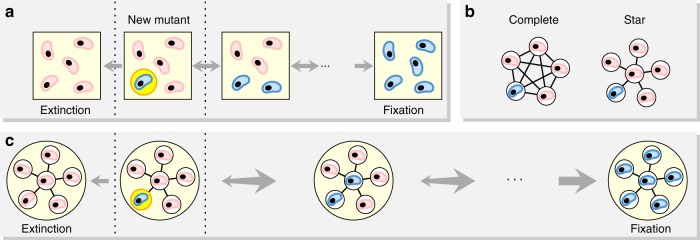
Moran process on graphs. **a** A new mutant (blue) appears in a population of finite size. The lineage of the new mutant can either become extinct or reach fixation. The Moran process is a birth–death process; in any one time step one new offspring is generated and one individual dies. **b** All fixed spatial structures can be described by graphs. The classical, well-mixed population corresponds to a complete graph, where all positions are equivalent. The star graph is a well-studied example of extreme heterogeneity, where one individual, the center, is connected to all others, but each leaf is only connected to the center. **c** Population structure influences both the fixation probability and the fixation time. An advantageous mutant introduced at a random vertex of a star graph is more likely to fixate than on a complete graph (the arrows pointing to the right are thicker), but the (average) fixation time on the star graph is much longer than on the complete graph (the arrows are longer). The star graph achieves amplification at the cost of deceleration

A classical and well-studied evolutionary process is the discrete-time Moran birth-death process^6^. Given a population of *N* individuals, at each time step, an individual is chosen for reproduction proportionally to its fitness; then the offspring replaces a random individual (see Fig. 1a). In the case of a well-mixed population, each offspring is equally likely to replace any other individual. For a single new mutant with relative fitness *r*, its fixation probability is *ρ* = (1 − 1/*r*)/(1 − 1/*r*^*N*^). Thus, for *r* > 1 and large *N* we have *ρ* ≈ 1 − 1/*r*^3,7^.

For measuring time, there are two natural options. The absorption time is the average number of steps of the Moran process until the population becomes homogeneous, regardless of whether the mutant fixates or becomes extinct. Alternatively, the (conditional) fixation time is the average number of steps of those evolutionary trajectories that lead to the fixation of the mutant, ignoring trajectories that lead to the extinction of the mutant. Since the evolutionary trajectories leading to extinction are typically shorter than those leading to fixation, the fixation time tends to be longer than the absorption time. Therefore, in our results concerning time we present lower bounds on the absorption time and upper bounds on the fixation time.

For the well-mixed population, both the absorption time and the fixation time are of the order of *N* log *N*^8,9^. Specifically, for *r* > 1 and large *N*, the absorption time is approximately [(*r* + 1)/*r*]*N* log *N* while the fixation time is approximately [(*r* + 1)/(*r* − 1)]*N* log *N*. For neutral evolution, *r* = 1, the absorption time is approximately *N* log *N* while the fixation time is (*N* − 1)^2^.

Both the fixation probability and the fixation time depend on population structure^10–18^. Evolutionary graph theory is a framework to study the effect of population structure. In evolutionary graph theory, the structure of a population is represented by a graph^7,19–23^: each individual occupies a vertex; the edges represent the connections to neighboring sites where a reproducing individual can place an offspring. The edge weights represent the proportional preference to make such a choice. The well-mixed population is given by the complete graph *K*_*N*_ where each individual is connected to each other individual (Fig. 1b). Graphs can also represent deme structured populations, where islands are represented by complete graphs and connections (of different weights) exist between islands. Graphs can also represent spatial lattices or asymmetric structures.

A well-studied example is the star graph *S*_*N*_, which has one central vertex and *N* − 1 surrounding vertices each connected to the central vertex (Fig. 1b). For the star graph, the fixation probability tends to approximately 1 − 1/*r*^2^ for *r* > 1 and large *N* while both the absorption and the fixation time is of the order of *N*^2^ log *N*^24–26^. Hence, if a mutant has 10% fitness advantage, which means *r* = 1.1, the star graph amplifies the advantage to 21%, but at the cost of increasing the time to fixation (Fig. 1c).

Several population structures have been identified that alter the fixation probability of advantageous mutants. Structures that decrease the fixation probability are known as suppressors of selection and those that increase it are known as amplifiers of selection^7,27–29^. However, amplification is usually achieved at the cost of increasing fixation time compared to the well-mixed population^13,17,30,31^. For example, the star graph has higher fixation probability but also longer fixation time as compared to the well-mixed population. There also exist superamplifiers (also known as arbitrarily strong amplifiers of natural selection) that guarantee fixation of advantageous mutants in the limit of large population size^32–35^. But those structures tend to require even longer fixation times.

We can refer to population structures that decrease the fixation time with respect to the well-mixed population as accelerators. Both the fixation probability and the fixation time play an important role in the speed of evolution. Ideally, we prefer a population structure that is both an amplifier and an accelerator, but all known amplifiers achieve amplification at the cost of deceleration. In fact, this slowdown can be so prominent that it outweighs the amplification and leads to longer evolutionary timescales^17^.

Here we show that absorption time on any amplifier is asymptotically at least as large as both the absorption and the fixation time on the well-mixed population. Given this negative result, we proceed to study the tradeoff between fixation probability and time more closely. We have computed fixation probabilities and fixation times for a large class of graphs. While within this class, the well-mixed population is optimal with respect to fixation time, and the star graph is favorable with respect to fixation probability, there is a very interesting tradeoff curve between fixation probability and fixation time. In other words, there exist population structures which provide different tradeoffs between high fixation probability and short fixation time. As our main analytical results, we present population structures that asymptotically achieve fixation probability equal to that of star graphs and fixation time similar to that of well-mixed populations. Thus, we achieve amplification with negligible deceleration. Finally, while the above analytical results are established for large population sizes, we also study evolutionary processes on population structures of small or intermediate size by numerical simulation. Specifically, we consider the effective rate of evolution as proposed by Frean, Rainey, and Traulsen^17^. Generally speaking, the well-mixed population has a high effective rate of evolution if the mutation rate is high, while the star graph has a high effective rate of evolution if the mutation rate is very low. We show that for a wide range of intermediate mutation rates, our new structures achieve higher effective rate of evolution than both the well-mixed population and the star graph.

## Results

We study several fundamental questions related to the probability-time tradeoff of a single advantageous mutant in a population of size *N*. Mutants can arise either spontaneously or during reproduction. Mutants that arise spontaneously appear at a vertex chosen uniformly at random among all *N* vertices. This is called uniform initialization. Mutants that arise during reproduction appear at each vertex proportionally to its replacement rate, which is called temperature of that vertex. This is called temperature initialization. We study the probability-time tradeoff for both types of initialization.

### Amplifiers and accelerators

First, we investigate whether there are population structures that are amplifiers and asymptotic accelerators of selection as compared to the complete graph (well-mixed population). We show that for any amplifier with population size *N*, the absorption time is of the order of at least *N* log *N*, for both types of initialization. Since the fixation time tends to be even longer than the absorption time and the fixation time for the complete graph is also of the order of *N* log *N*, regardless of initialization, this suggests that no amplifier is an asymptotic accelerator. Moreover, we show that the same conclusion holds for graphs that decrease the fixation probability by not more than a constant factor. Our result does not completely exclude the possibility of population structures with absorption time asymptotically shorter than that of the complete graphs but it shows that, if such structures do exist, then the fixation probability has to tend to 0 as the population size *N* grows large. While the above results holds in the limit of large population size, we present a small directed graph that is a suppressor and has a slightly shorter fixation time than the complete graph for the same population size (see Supplementary Note 1, Section 3.1).

### Tradeoff under uniform initialization

Second, we consider uniform initialization. There are two interesting questions: First, for fixed (small) population size, how do different population structures fare with respect to the probability-time tradeoff? Second, in the limit of large population size, do there exists population structures that achieve the same amplification as the star graph, with shorter fixation time? Our results are as follows.

**Fig. 2:**
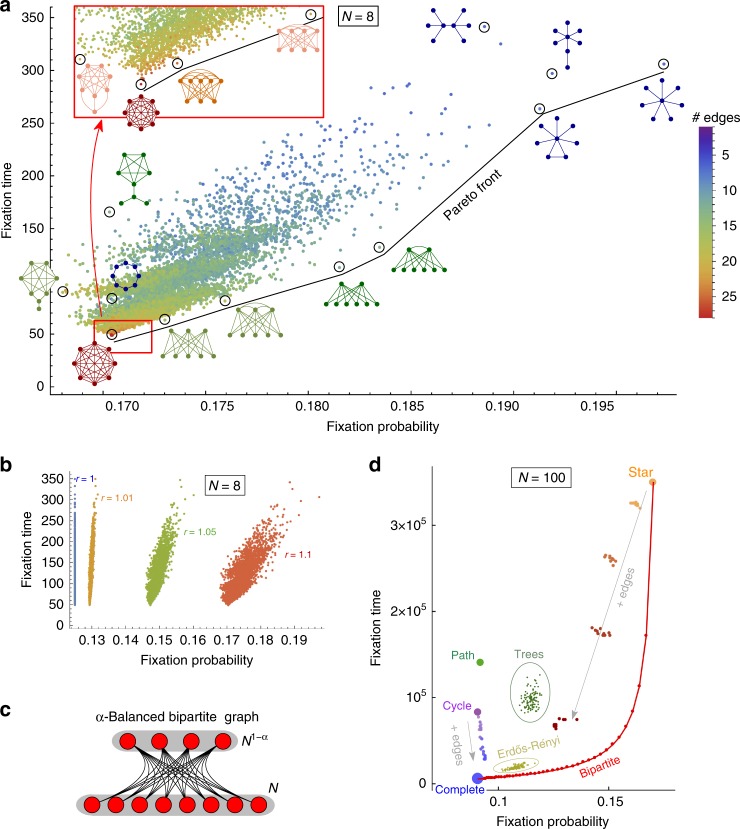
Fixation probability and time under uniform initialization. **a** Numerical solutions for all 11,117 undirected connected graphs of size *N* = 8 (see Supplementary Fig. 1 for 2.3 · 10^5^ graphs of size *N* = 9). Each graph is represented by a dot and color corresponds to the number of its edges. The *x*- and *y*-coordinates show the fixation probability and the fixation time for a single mutant with relative fitness *r* = 1.1, under uniform initialization. The graphs to the right of the complete graph are amplifiers of selection: they increase the fixation probability. Any graph below the complete graph would be an accelerator of selection: it would decrease the fixation time. Graphs close to the bottom right corner provide good tradeoff between high fixation probability and short fixation time. **b** Similar data for varying *r*. Under uniform initialization, the fixation probability of a neutral mutant equals 1/*N*, independent of the graph structure. As *r* approaches 1, the point cloud gets closer to a vertical line. **c** An *α*-Balanced bipartite graph *B*_*N*,*α*_ is a complete bipartite graph with *N* vertices in the larger part and *N*^1−*α*^ vertices in the smaller part. Here *N* = 8 and *α* = 1/3. We prove that for large *N*, the *α*-Balanced bipartite graphs achieve the fixation probability of a star graph and approach the fixation time of the complete graph. **d** Computer simulations for selected graphs of size *N* = 100 such as Trees, random Erdős–Rényi graphs, and Cycles or stars with several extra edges (see Supplementary Note 1, Section 4 for details). Bipartite graphs provide great tradeoffs between high fixation probability and short fixation time

First, for small population size, both fixation probability and fixation time can be computed numerically^36^. We do this for all graphs with *N* = 8 vertices and a mutant with relative fitness advantage *r* = 1.1 (see Fig. 2a). We observe that the complete graph has the shortest fixation time, and the star graph has the highest fixation probability. However, the star graph has much longer fixation time than the complete graph. While some graphs have smaller fixation probability and longer fixation time than the complete graph, there are other graphs which provide a tradeoff between high fixation probability and short fixation time. In particular, there are Pareto-optimal graphs. Recall that in two- or multi-dimensional optimization problems, the Pareto front is the set of non-dominated objects. In our case, the Pareto front consists of graphs for which the fixation probability can not be improved without increasing the fixation time. For *N* = 8 and *r* = 1.1 the complete graph and the star graph are the two extreme points of the Pareto front. This finding holds for other values of *r* > 1 as well (Fig. 2b).

We answer the second question in the affirmative. The tradeoff results (Fig. 2a) that we study allow us to obtain graphs which we call α-Balanced bipartite graphs. Intuitively, they are defined as follows: we split the vertices into two groups such that one is much smaller than the other, but both are relatively large. Then we connect every two vertices that belong to different groups. We show that, in the limit of large population size, this bipartite graph achieves the same fixation probability as the star graph and that its fixation time asymptotically approaches that of the complete graph. Formally, an *α*-Balanced bipartite graph *B*_*N*,*α*_ is a complete bipartite graph with the parts containing *N* and *N*^1−*α*^ vertices (see Fig. 2c for illustration with *N* = 8 and *α* = 1/3). We show that the fixation probability of such graphs tends to 1−1/*r*^2^ while the fixation time is of the order of *N*^1+*α*^ log *N*, for any *α* > 0 (compared to *N* log *N* fixation time of complete graph). Thus we achieve the best of two worlds, that is, we present a graph family that, in the limit of large population size, is as good an amplifier as the star graph and almost as good with respect to time as the complete graph. As a byproduct, we prove that on a star graph, both the absorption and the fixation time are of the order of *N*^2^ log *N* for any fixed *r* > 1 which is in alignment with known bounds and approximation results^25,26^.

Moreover, we support the analytical result with computer simulations for fixed population size *N* = 100. We compute the fixation probability and time for selected families of graphs, such as Trees or random Erdős–Rényi graphs (see Fig. 2d). The *α*-Balanced bipartite graphs outperform all of them. Hence the analytical results are interesting not only in the limit of large population but already for relatively small population sizes.

### Tradeoff under temperature initialization

Third, we consider temperature initialization. The above questions for uniform initialization are also the relevant questions for temperature initialization. Our results are as follows.

**Fig. 3:**
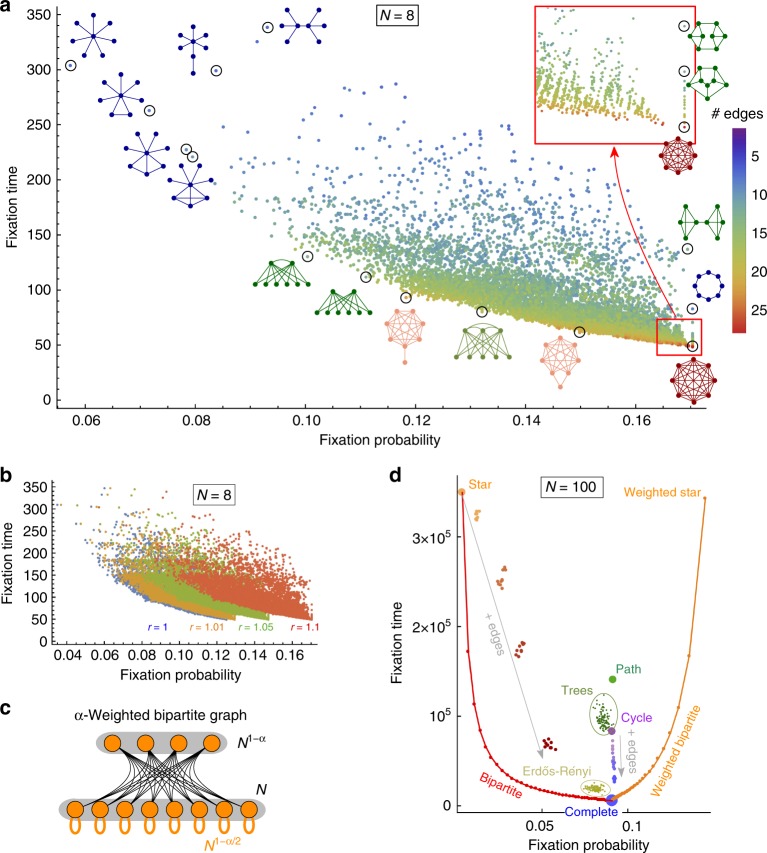
Fixation probability and time under temperature initialization. **a** Numerical solutions for all undirected connected graphs of size *N* = 8, under temperature initialization (*r* = 1.1). There are no amplifiers and no (strict) accelerators. By the isothermal theorem^7^, all the regular graphs achieve the same fixation probability as the complete graph. **b** Similar data for varying *r*. **c** The *α*- Weighted bipartite graphs are obtained by adding self-loops with large weight to all vertices in the larger part of an *α*-Balanced bipartite graph. We prove that for large *N*, the *α*-Weighted bipartite graphs improve the fixation probability to 1 − 1/*r*^2^ and approach the fixation time of a complete graph. **d** Computer simulations for selected graphs of size *N* = 100. It is known than among unweighted graphs, only a very limited amplification can be achieved^35^. Our *α*- Weighted bipartite graphs (with self-loops of varying weight) overcome this limitation and provide tradeoffs between high fixation probability and short fixation time

First, we again numerically compute the fixation probability and the fixation time for all graphs with *N* = 8 vertices (see Fig. 3a). In contrast to the results for uniform initialization (Fig. 2a), under temperature initialization, the complete graph has both the highest fixation probability and the shortest fixation time. This is the case for other *r* > 1 too (see Fig. 3b).

Second, we present analytical results. Figure 3a shows that there is no tradeoff for temperature initialization. The result is not surprising as it has recently been shown that, for temperature initialization, no unweighted graphs can achieve substantial amplification^35^, and in the present work we have established that the complete graph is asymptotically optimal among amplifiers with respect to absorption time. Thus, the relevant analytical question is whether weighted graphs can achieve interesting tradeoffs between fixation probability and time. We answer this question in the affirmative by presenting a weighted version of *α*-Balanced bipartite graphs (see Fig. 3c). Intuitively, we add weighted self-loops to all vertices in the larger group of an *α*-Balanced bipartite graph, such that when such a vertex is selected for reproduction, its offspring replaces the parent most of the time and migrates to the smaller group only rarely. Formally, the *α*- Weighted bipartite graph *W*_*N*,*α*_ is a complete bipartite graph with the parts containing *N* and *N*^1−*α*^ vertices. Moreover, each vertex in the larger part has an extra self-loop of weight approximately *N*^1−*α*/2^. We show that, in the limit of large population size, this weighted bipartite graph structure achieves fixation probability 1 − 1/*r*^2^ (which is the same as the star graph under uniform initialization), while the fixation time is of the order of *N*^1+*α*^ log *N*, for any *α* > 0 (compared to *N* log *N* fixation time of complete graph). Thus we again achieve the best of two worlds, that is, we present a graph family that, in the limit of large population, is as good an amplifier as the star graph (under uniform initialization) and almost as good with respect to time as the complete graph.

Moreover, as before, Fig. 3d shows computer simulations for *N* = 100, including Trees, random Erdős–Rényi graphs and the Bipartite graphs. The *α*- Weighted bipartite graphs are the only graphs that considerably increase the fixation probability as compared to the complete graph.

### Effective rate of evolution

Finally, we study the effectiveness of the presented population structures for small population sizes. We use an elegant mathematical formula for the effective rate of evolution that combines both fixation probability and fixation time^17^. Let $t_1 = \frac{1}{{N\mu \rho }}$ denote the expected number of generations to generate a mutant that eventually fixates, where *N* is the population size, *μ* is the mutation rate, and *ρ* is the fixation probability. Let $t_2 = \frac{\tau }{N}$ denote the expected number of generations for a mutant to fixate once it is generated. Note that *τ* is the fixation time measured in steps of the Moran process, and $\frac{\tau }{N}$ represents the number of generations. The effective rate of evolution is defined as the inverse of the sum of the above two quantities, i.e., $\frac{1}{{t_1 + t_2}}$. The effective rate of evolution was studied for the complete graph and for the star graph under uniform initialization^17^. Here we further investigate the effective rate of evolution for *α*-Balanced bipartite graph under uniform initialization, and for *α*-Weighted bipartite graphs under temperature initialization, for relatively small population sizes.

**Fig. 4:**
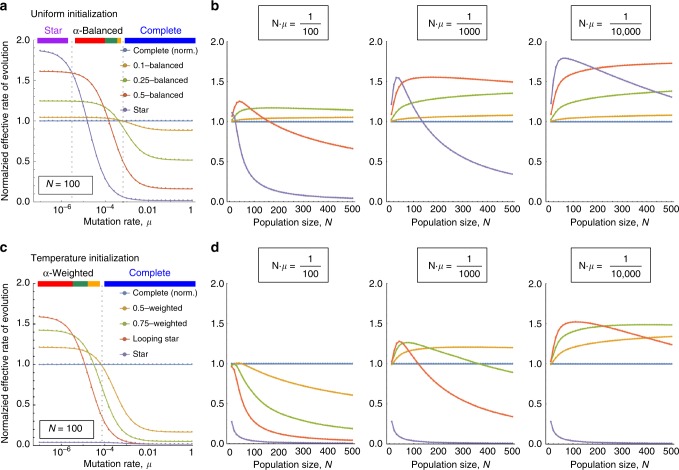
Effective rate of evolution. The effective rate of evolution depends on the population size, *N*, the mutation rate, *μ*, and the population structure. For uniform initialization, we compare five different population structures: the complete graph (blue), *α*-Balanced graphs with *α* ∈ {0.1, 0.25, 0.5} (orange, green, red), and the star graph (purple), always showing the relative rate of evolution with respect to the complete graph. **a** We fix *N* = 100, *r* = 1.1, and vary *μ* = 10^−7^, …, 10^0^. The complete graph has a higher effective rate of evolution if the mutation rate is high (*μ* > 10^−3^) and star graph is favorable if the mutation rate is low (*μ* < 3 · 10^−6^). In the intermediate regime, suitable *α*-Balanced graphs outperform both of them. **b** We fix *r* = 1.1 and *N*⋅*μ* ∈ {10^−2^, 10^−3^, 10^−4^} and vary *N* = 10, 20, …, 500. The star graph is favorable if mutations are rare (*N*⋅*μ* = 10^−4^ and *N* small) and the complete graph is favorable if mutations are common (*N* ⋅ *μ* ≥ 10^−1^). In the intermediate regime, suitable *α*-Balanced graphs are more efficient. **c**, **d** Analogous data for temperature initialization. This time we compare the complete graph (blue) and the star graph (purple) with *α*-Weighted bipartite graphs for *α* ∈ {0.25, 0.5, 1} (orange, green, red). As before, the complete graph dominates if mutations are common. In other cases, *α*-Weighted bipartite graphs are preferred. The star graph is not an amplifier for temperature initialization

Regarding uniform initialization, we compute the effective rate of evolution on *α*-Balanced bipartite graphs for a wide range of mutation rates *μ* and compare it to the effective rate of evolution on complete graphs and star graphs (see Fig. 4a for fixed population size and Fig. 4b for varying population sizes). The complete graph is more effective for high mutation rates and the star graph is more effective for low mutation rates but in the intermediate regime, suitable *α*-Balanced bipartite graphs are more effective than both the complete graph and the star graph. This is in a perfect alignment with the Pareto front presented in Fig. 2a.

Regarding temperature initialization, we study *α*-Weighted bipartite graphs instead of *α*-Balanced bipartite graphs (Fig. 4c, d). As before, the complete graph is the most effective population structure for high mutation rates. However, the star graph is a suppressor under temperature initialization and performs poorly. Therefore, except for the high mutation rate regime, various *α*-Weighted bipartite graphs achieve higher effective rate of evolution than both the complete graph and the star graph.

## Discussion

Many previous studies have explored how population structure affects the fixation probability of new mutants^7,16,19–21,23,32–35,37,38^. While such studies cover one major aspect of evolutionary dynamics, the other aspect, which is fixation time, is much less studied. Both fixation probability and fixation time play an important role in determining the rate of evolution. If the mutation rate is low, the rate-limiting step is waiting for an advantageous mutant to occur. In this regime the fixation probability is more important than the fixation time. Conversely, if the mutation rate is high, then fixation time is more relevant than fixation probability. In the intermediate-mutation rate regime, the tradeoff between fixation probability and fixation time must be considered. We study this tradeoff and propose population structures, called *α*-Balanced bipartite graphs and *α*-Weighted bipartite graphs, that provide substantial amplification with negligible increase in the fixation time. This is in stark contrast with all previous works that achieve amplification at the cost of asymptotically increasing the fixation time. As a consequence, compared to previous works, our population structures enable higher effective rate of evolution than the well-mixed population for a wide range of mutation-rate regimes.

There are some interesting mathematical questions that remain open. While we show that (i) amplifiers cannot have better asymptotic absorption time than the well-mixed population (in the limit of large population size, *N* → ∞), and (ii) there are graphs of fixed population size *N*, that are suppressors and have shorter fixation time than the well-mixed population, two particularly interesting questions are: (a) Does there exist an amplifier of fixed population size that has shorter fixation time than the well-mixed population? (b) Does there exist a graph family (which must be suppressing) that has better asymptotic fixation time (for *N* → ∞) than the well-mixed population?

Note that, in general, clonal interference can occur and the fixation of a mutant need not be achieved before the next mutation arrives^4,39,40^. Thus, the fixation probability and fixation time alone may not completely characterize the performance of a population structure with respect to the overall rate and efficiency of an evolutionary search process. Nevertheless, the effective rate of evolution and the probability-time tradeoff curves are indicative of the efficacy of each population structure in speeding-up evolution. The numerical and experimental study of population structures in the presence of clonal interference is another interesting direction for future work.

The population structures which we have described here could become an important tool for in vitro evolution^41–44^, since they can substantially speed up the process of finding advantageous mutants. In vitro evolution, can be used to discover optimized protein or nucleotide sequences for any medical or industrial purpose. Depending on the mutation-rate regime, our work shows that different population structures can lead to more effective time scales of discovery.

## Methods

Here we present details of the model and sketches of the formal proofs of our results, pointing to the relevant parts of the Supplementary Note 1 for details.

### Uniform and temperature initialization

Given a graph *G* with *N* vertices and one specific vertex *v*, we denote by *ρ*(*G*, *v*, *r*) the fixation probability of a single mutant with fitness *r* starting at vertex *v*, in a standard Moran birth-death process. Then the fixation probability under uniform initialization is simply the average $\rho (G,{\Bbb U},r) = \frac{1}{N}\mathop {\sum}\nolimits_v \rho (G,v,r)$. The fixation probability under temperature initialization is a weighted average $\rho (G,{\Bbb T},r) = \mathop {\sum}\nolimits_v t (v)\cdot \rho (G,v,r)$, where *t*(*v*) is the temperature of vertex *v*. The temperature (turnover rate) of vertex *v* is defined by $t(v) = \frac{1}{N}\mathop {\sum}\nolimits_u w (u,v)$, where *w*(*u*, *v*) is the probability that an individual reproducing at vertex *u* places offspring at vertex *v*.

### Amplifiers and accelerators

We prove that for any graph *G* on *N* vertices, the absorption time is greater than $\frac{{\rho (G,r)}}{r}N\,{\mathrm{log}}\,N$. Specifically, this shows that the absorption time is of the order of at least *N* log *N* for the complete graph, for any amplifier, and for any weak suppressor (a graph that decreases the fixation probability by not more than a constant factor). In the proof, we consider the Markov chain corresponding to the Moran process. Briefly, for every *k* = 1, …, *N* − 1, we denote by $t_k^X$ the expected time it takes to gain a single mutant from any configuration *X* consisting of *k* mutants. To gain a mutant, one of the *k* mutants has to be selected for reproduction (and then the mutant has to replace a resident). We show that if the probability of fixation is at least a constant, then this yields a lower bound for *t*_*k*_ that is proportional to *N*/*k*. Summing over all *k*’s we get that the total fixation time is of the order of at least *N*/1 + *N*/2 + … + *N*/(*N* − 1) ≈ *N* log *N*. Since the absorption time for the complete graph is also proportional to *N* log *N*, no amplifier or a weak suppressor is asymptotically faster than the complete graph in terms of absorption time. We remark that this does not imply that the complete graph is faster than any other graph of the same size. In fact, this is not true: We present a simple (directed) suppressor whose fixation time is shorter than the fixation time on a complete graph of the same size. See Supplementary Note 1, Section 3.1 for details.

### Tradeoff under uniform initialization

Under uniform initialization, we consider *α*-Balanced bipartite graphs *B*_*N*,*α*_ with parts of sizes *N* and *N*^1−*α*^, where *α* > 0 is fixed. We prove that the fixation probability of such graphs *B*_*N*,*α*_ tends to 1 − 1/*r*^2^ as the population size *N* tends to infinity. To that end, we use a general formula for the fixation probability on a complete bipartite graph^45^ and compute the limit when the size of the smaller part is equal to *N*^1−*α*^. Next, we argue that the fixation time for *α*-Balanced bipartite graphs *B*_*N*,*α*_ is of order approximately *N*^1+*α*^ · log *N* which is asymptotically less than *N*^2^·log *N* for the star graph. We confirm this by providing matching numerical results (see Supplementary Figs. 2 and 3). Intuitively, during evolutionary dynamics on a star graph, the non-central vertices of the star evolve like a well-mixed population except that an evolutionary step occurs only if the vertex picked for reproduction was the center of the star. This happens approximately once in every *N* steps, hence the star graph is approximately *N*-times slower than the well-mixed population. Similarly, for *α*-Balanced bipartite graph the larger part evolves like a well-mixed population, except that an evolutionary step occurs only if the vertex picked for reproduction belonged to the smaller part. Hence, compared to the star graph, we get a speed up by a factor of *N*^1−*α*^, for the total fixation time *N*^2^ log *N*/*N*^1−*α*^ = *N*^1+*α*^ log *N*. See Supplementary Note 1, Section 3.2 for details.

### Tradeoff under temperature initialization

Under temperature initialization, we define *α*- Weighted bipartite graphs *W*_*N*,*α*_ with parts of sizes *N* and *N*^1−*α*^ and self-loops of weight *N*^1−*α*/2^ − *N*^1−*α*^ at each vertex of the larger part. We closely follow the arguments for the *α*-Balanced bipartite graphs, and prove that, in the limit of large population size, the fixation probability of such *α*- Weighted bipartite graphs *W*_*N*,*α*_ tends to 1 − 1/*r*^2^ and that the fixation time is of order approximately *N*^1+*α*^ log *N*. See Supplementary Note 1, Section 3.3 for details.

### Effective rate of evolution

Given a mutation rate *μ* and a population of *N* individuals placed on a population structure where a new mutant has fixation probability *ρ* and fixation time *τ*, the *effective rate of evolution* is given by $\frac{1}{{t_1 + t_2}}$, where $t_1 = \frac{1}{{N\mu \rho }}$ is the expected number of generations to produce a mutant that eventually fixates and $t_2 = \frac{\tau }{N}$ is the expected number of generations for the mutant to fixate once it was generated. This is closely based on a notion introduced before^17^ but with both *t*_1_ and *t*_2_ measured in generations. We numerically compute the effective rate of evolution for various population structures (complete graph, star graph, *α*-Balanced bipartite graphs, and *α*-Weighted bipartite graphs), population sizes, and mutation rates and compare the results (see Supplementary Fig. 4 for additional regimes).

### Reporting summary

Further information on experimental design is available in the Nature Research Reporting Summary linked to this article.

## Supplementary information

Supplementary material

Reporting Summary

## Data Availability

The datasets generated during and/or analyzed during the current study and the related computer code are available at https://figshare.com/s/38f0d4ceffb32ce49bc4.

## Code availability

Computer code used in this study is available at https://figshare.com/s/38f0d4ceffb32ce49bc4.
