# Supplementary material for: Population structure determines the tradeoff between fixation probability and fixation time
Source: Commun Biol. 2019 Apr 23;2:138. doi: 10.1038/s42003-019-0373-y (PMC6478818; doi:10.1038/s42003-019-0373-y)
Supplement: Supplementary file 1 — Supplementary material [file 42003_2019_373_MOESM1_ESM.pdf]

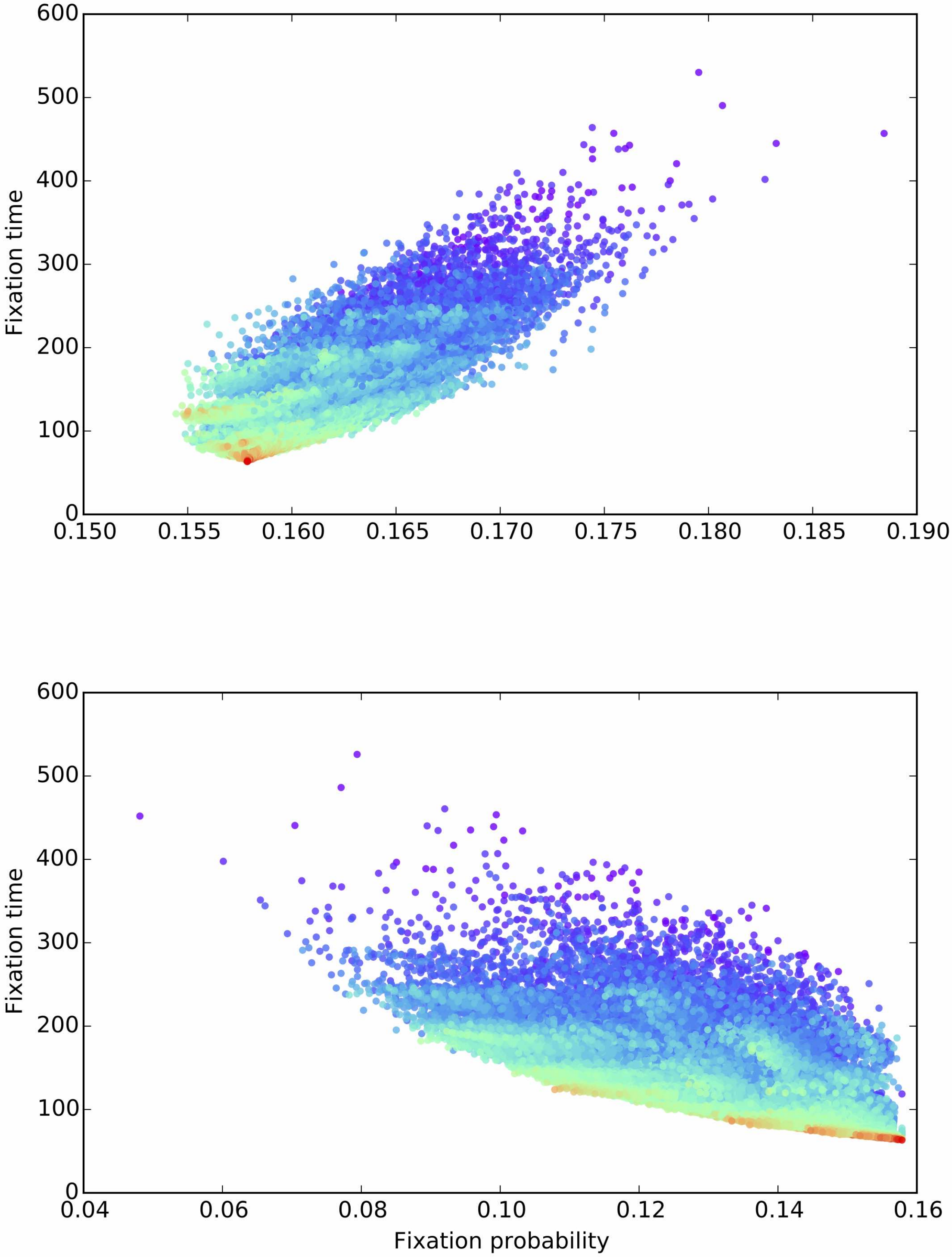

Supplementary Figure 1: All graphs with $N = 9$ vertices. Similarly to Figures 2a and 3a, we show the fixation probability and time for all 261 080 connected graphs with $N = 9$ vertices and $r = 1.1$ under uniform (top panel) and temperature (bottom panel) initialization. Here $r = 1.1$. The results are qualitatively the same as for $N = 8$.

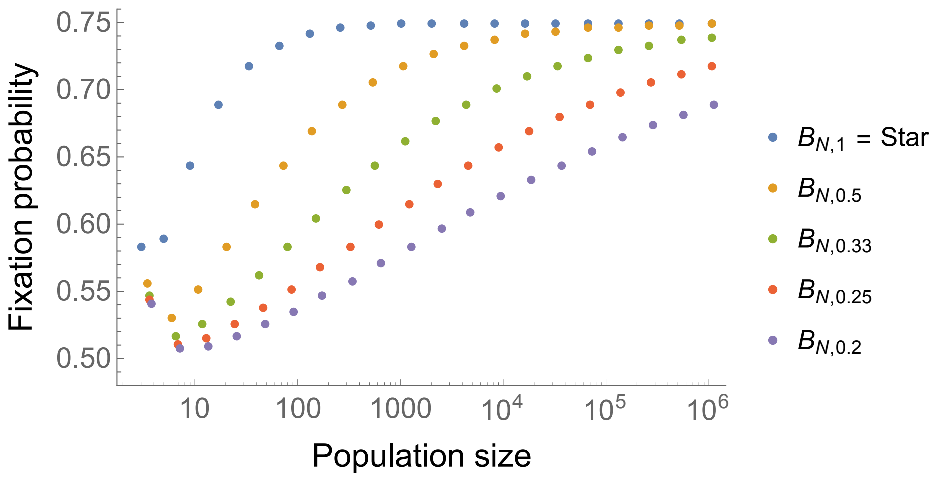

Supplementary Figure 2: Fixation probability on bipartite graphs $B_{N,\alpha}$**.** We fix $r = 2$ and consider the α-Balanced bipartite graphs for $\alpha = 1$ (i.e. a star) and $\alpha \in \{0.5, 0.33, 0.25, 0.2\}$. The dots are exact values of the fixation probability under uniform initialization, computed by numerically solving large systems of linear equations. The figure illustrates that the fixation probability tends to $1 - 1/r^2$ for any positive $\alpha > 0$.

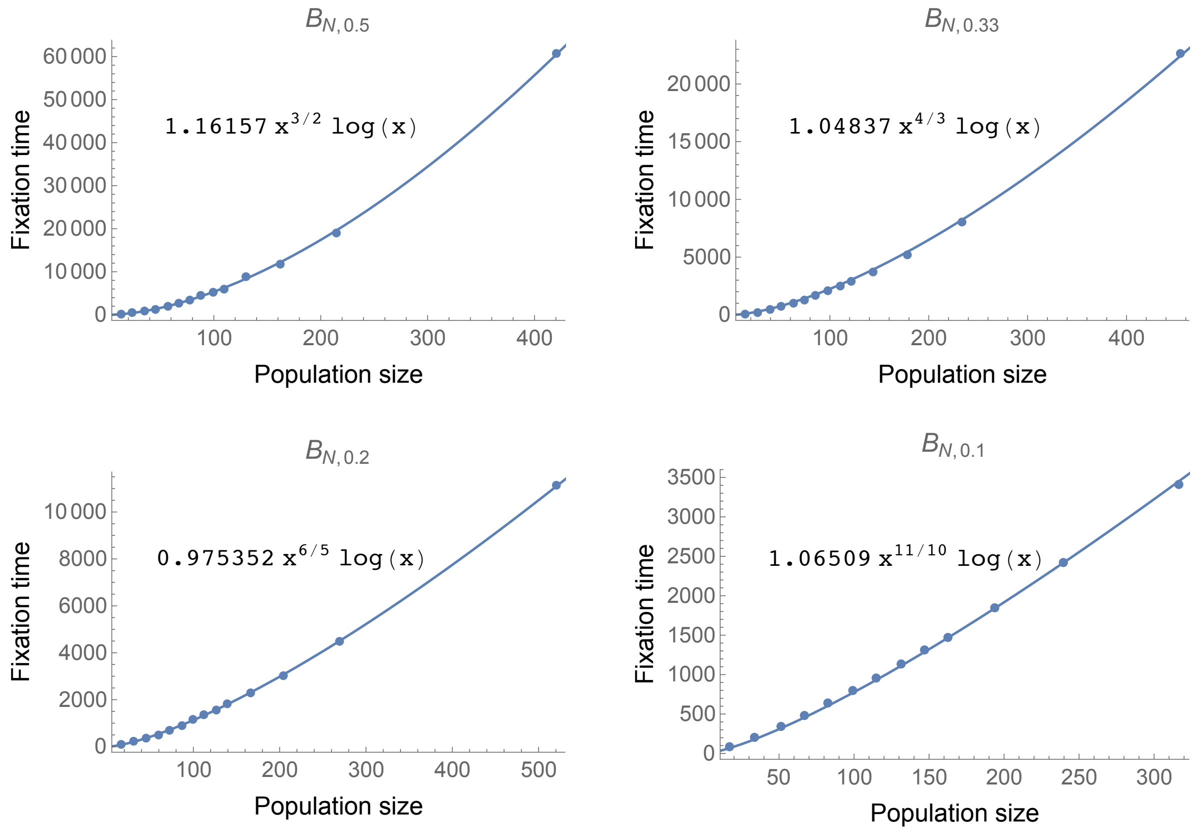

Supplementary Figure 3: Fixation time on bipartite graphs $B_{N,\alpha}$. We fix $r = 2$ and consider the α-Balanced bipartite graphs for $\alpha \in \{0.5, 0.33, 0.2, 0.1\}$ and for N up to 500. The dots are exact numerical solutions, the lines are the best fits. The figure suggest that the correct asymptotics for the fixation time $T(B_{N,\alpha})$ is $\Theta(N^{1+\alpha} \log N)$ for any $\alpha \in (0, 1]$.

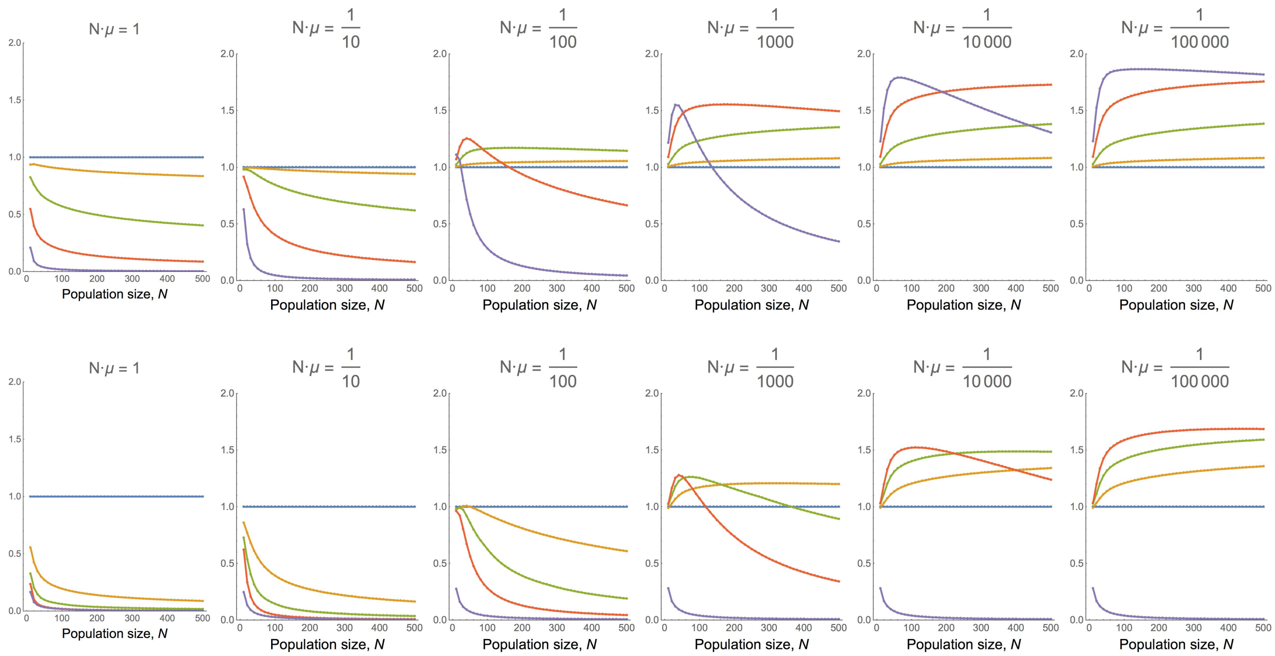

Supplementary Figure 4: Normalized effective rate of evolution for more regimes. As in the Figure 4 from the main text, we fix $r = 1.1$ and $N \cdot \mu = 10^{-i}$ for $i = 0, \ldots, 5$ and vary population size $N = 10, 20, \ldots, 500$. In the first row, we consider uniform initialization and the following graphs: Complete graph (blue), α-Balanced graph for $\alpha \in \{0.1, 0.25, 0.5\}$ (orange, green, red), and the Star graph (purple) which is the same as a 1-Balanced graph. In the second row, we consider temperature initialization and the following graphs: Complete graph (blue), Star graph (purple), and α-Weighted bipartite graphs for $\alpha \in \{0.25, 0.5\}$ (orange, green) and Weighted Star ($\alpha = 1$, red).

Supplementary Note 1: Population structure determines the tradeoff between fixation probability and fixation time

Josef Tkadlec[a, 1], Andreas Pavlogiannis[b, 1], Krishnendu Chatterjee[a], and Martin A. Nowak[c, ⋆]

[a]IST Austria, A-3400 Klosterneuburg, Austria
[b]Lab for Automated Reasoning and Analysis, EPFL, CH-1015 Lausanne, Switzerland
[c]Program for Evolutionary Dynamics, Department of Organismic and Evolutionary Biology, Department of Mathematics, Harvard University, Cambridge, MA 02138, USA
[1]J.T. and A.P. contributed equally to this work.
[⋆]To whom correspondence should be addressed. E-mail: martin_nowak@harvard.edu

Contents

1 Organization **5**

2 Model and Overview **6**
2.1 Moran process on graphs 6
2.2 Notation for asymptotic behavior 7
2.3 Graphs 7
2.4 Overview of theoretical results 8

3 Formal proofs of theoretical results **9**
3.1 Lower bound on absorption time 9
3.2 α-Balanced bipartite graphs 10
3.3 Weighted bipartite graphs 17
3.4 Time on Complete graph 18

4 Details of figure legends **22**

5 Future research directions **22**

1 Organization

The organization of this text is as follows. In the next section, we formally introduce the model of Moran Birth-death process on a graph and the graph families we later analyze in detail. Then we summarize our theoretical results. In Section 3 we present formal proofs for the theoretical results. In Section 4 we present the details of the numerical and computer simulation results presented in the main paper. In Section 5 we state several interesting directions for future research.

2 Model and Overview

2.1 Moran process on graphs

Moran Birth-death process is a discrete-time stochastic (random) process that models evolutionary dynamics in a spatially structured population. The population structure is represented by a connected graph G, possibly with weighted edges and/or self-loops. At all times, each vertex of the graph is occupied by a single individual that is of one of two types: either a *resident* or a *mutant*. The individuals of one type are considered indistinguishable. Moreover, residents are assigned (normalized) fitness 1 while the mutants have fitness r. Here we consider advantageous mutants ($r > 1$). In one step of the process, an individual is selected for reproduction randomly and proportionally to its fitness. This individual produces an offspring that is a copy of itself. This offspring then selects one of the adjacent edges proportionally to the edge weight and travels along that edge to replace the individual at its other endpoint. (If the selected edge happened to be a self-loop then the offspring replaces the parent and nothing changes.) These steps continue until the population becomes homogeneous: either all individuals are mutants (*fixation* occurred) or they are all residents (*extinction* occurred). The well-mixed population is modelled by an unweighted complete graph (without self-loops).

Initialization scheme. We study the situation of a single mutant invading a population of residents. This initial mutant can appear either spontaneously or during reproduction. In the first case, called *uniform initialization*, the mutant is placed at a vertex chosen uniformly at random. In the second case, called *temperature initialization*, we perform one step of the Moran process in a population that consists entirely of residents and place the mutant at the vertex that the offspring migrates to. Formally, the mutant is placed at a random vertex, proportionally to the temperature (or turnover rate) of that vertex. Here temperature $t(v)$ of a vertex v is defined by

$$t(v) = \sum_{u \in N(v)} \frac{w(u,v)}{\sum_{v' \in N(u)} w(u,v')},$$

where $w(u,v)$ is the weight of edge between u and v and $N(v)$ is the set of *neighbors* of v, that is vertices connected to v by an edge.

Fixation probability and time. Given a graph G and relative fitness advantage r, let $\mathrm{fp}(G,r)$ be the fixation probability of a single mutant under uniform initialization, and let $\mathrm{fp}_{\mathcal{T}}(G,r)$ stand for temperature initialization. Similarly, we define $T(G,r)$ (or $T_{\mathcal{T}}(G,r)$) to be the fixation time, that is the expected number of steps of the Moran process until the mutants reach fixation (conditioning on them doing so). Likewise we define $\mathrm{ET}(G,r)$ (or $\mathrm{ET}_{\mathcal{T}}(G,r)$) to be the extinction time and $\mathrm{AT}(G,r)$ (or $\mathrm{AT}_{\mathcal{T}}(G,r)$) to be the (unconditional) absorption time.

Amplifiers and superamplifiers. A graph G_N with N vertices is called an *amplifier* if it increases the fixation probability of any advantageous mutant, as compared to the Complete graph (that is, $\mathrm{fp}(G_N,r) > \mathrm{fp}(K_N,r)$ for any $r > 1$). A sequence of graphs $(G_N)_{N=1}^{\infty}$ of increasing size is called a *superamplifier* (or *arbiterarily strong amplifier*) if, in the limit of large N, it guarantees the fixation of any advantageous mutant (that is, $\mathrm{fp}(G_N,r) \to 1$ as $N \to \infty$).

2.2 Notation for asymptotic behavior

To talk about asymptotic behavior (in the limit of large population size N), we use standard mathematical notations $o(\cdot)$, $O(\cdot)$, and $\Theta(\cdot)$ that denote asymptotically strictly smaller, asymptotically less than or equal to, and asymptotically equal to (up to a constant factor), respectively. For example, we will write $\frac{1}{N} = o(1)$ ($\frac{1}{N}$ is much smaller than 1, for large N) or $\frac{1}{2}N(N+1) = \Theta(N^2)$. For detailed treatment see [1, Section 1.3]

2.3 Graphs

We introduce and study the following graphs.

Complete graph. Complete graph K_N on N vertices models a well-mixed population. This case is well understood. In particular, the fixation probability satisfies

$$\mathrm{fp}(K_N, r) = \mathrm{fp}_{\mathcal{T}}(K_N, r) = \frac{1 - 1/r}{1 - 1/r^N} \to 1 - 1/r$$

for $r > 1$ as $N \to \infty$ and the (unconditional) absorption time is of the order of $\Theta(N \log N)$ [2]. In fact, using a standard difference method one can derive that, for $r > 1$, we have $\mathrm{AT}(K_N, r) \approx \frac{r+1}{r} \cdot N \log N$ and $T(K_N, r) \approx \frac{r+1}{r-1} \cdot N \log N$. For reference purposes we present those proofs in Section 3.4.

Star graph. Star graph S_N consists of one central vertex connected to each of the remaining $N - 1$ vertices on the periphery. For large N, it is known that $\mathrm{fp}(S_N, r) \to 1 - 1/r^2$ and that the absorption and fixation time are of the order of at most $O(N^2 \log N)$ and $O(N^3)$, respectively [4]. In fact, as a corollary of our results on α-Balanced bipartite graph, we show that both the absorption time and the fixation time are of the order of $\Theta(N^2 \log N)$. The bottom line is that, under uniform initialization, the Star graph amplifies the fixation probability but at the cost of substantially increasing the fixation time.

α-Balanced bipartite graph. For uniform initialization we present a family of graphs that, in the limit of large population size, achieve the fixation probability of the Star graph and the fixation time almost as good as the Complete graph. The graphs are complete bipartite graphs with both parts large but one part asymptotically larger than the other one. Formally, given N and $\alpha \in (0, 1]$, the α-Balanced bipartite graph $B_{N,\alpha}$ is a complete bipartite graph with parts of size $N^{1-\alpha}$ and N. That is, there are $N^{1-\alpha}$ vertices in one part, N vertices in the other part, and all edges that connect vertices in different parts. The case $\alpha = 1$ corresponds to a Star graph.

Weighted bipartite graphs. For temperature initialization, the Star graph and the α-Balanced bipartite graphs fail to amplify. We present another family of weighted graphs with self-loops that, in the limit of large population size, provide fixation probability $1 - 1/r^2$ (the same as Star graph under uniform initialization) and the fixation time almost as good as the Complete graph. The graphs are obtained by adding self-loops of relatively large weight to all vertices in the larger part of an α-Balanced bipartite graph. Formally, given N and $\alpha \in (0, 1)$, the Weighted bipartite graph $W_{N,\alpha}$ is a complete bipartite graphs with one (smaller) part of size $N^{1-\alpha}$, one (larger) part of size N, and every vertex of the larger part having a self-loop of such a weight w that $N^{-\alpha/2} = \frac{N^{1-\alpha}}{w+N^{1-\alpha}}$. The case $\alpha = 1$ is closely related to a Looping Star [3].

2.4 Overview of theoretical results

Here we present the summary of our theoretical results. They are all related to the tradeoff between fixation probability and fixation time, under both uniform and temperature initialization.

First, we prove that no amplifier is asymptotically faster than the Complete graph in terms of absorption time (recall that $T(K_N, r) = \Theta(N \log N)$, see Section 3.4).

Theorem 1. *Fix $r > 1$. Let G be any graph with $N \geq 2$ vertices and let $p = \mathrm{fp}(G, r)$ be the fixation probability of a single mutant under uniform initialization. Then*

$$\mathrm{AT}(G, r) \geq \frac{p}{r} \cdot N \cdot H_{N-1},$$

where $H_{N-1} = \frac{1}{1} + \frac{1}{2} + \cdots + \frac{1}{N-1} \geq \log N$. In particular, $\mathrm{AT}(G, r) \geq \frac{p}{r} \cdot N \log N$ for an arbitrary graph G and $\mathrm{AT}(A, r) \geq \frac{r-1}{r^2} \cdot N \log N$ for an arbitrary amplifier A.

Second, we give tight results for the fixation time on Bipartite graphs. In particular, we prove that under uniform initialization, certain α-Balanced bipartite graphs $B_{N,\alpha}$ asymptotically achieve the fixation probability of the Star graph and the fixation time almost as good as the Complete graph. The analysis of fixation probability is relatively straightforward. For fixation time, we provide tight lower and upper bounds. We first present the lower bound that is proportional to $N^{1+\alpha} \log N$. For the upper bound we then distinguish two cases: If the size of the smaller part is small, that is $N^{1-\alpha} = o(\sqrt{N})$, then the argument is simpler and we get a matching upper bound. If the size of the smaller part is relatively close to N, the upper bound has an additional factor of N^{α}. As a consequence, we can prove the following theorem.

Theorem 2. *Fix $\alpha \in (0, 1]$ and $r > 1$. Let $B_{N,\alpha}$ be the α-Balanced bipartite graph. Then*

- $\mathrm{fp}(B_{N,\alpha}, r) \to 1 - 1/r^2$.
  - *(small center) If $\alpha \in (0.5, 1)$ then there exist constants c_1, c_2 such that*

    $$c_1 \cdot N^{1+\alpha} \log N \leq \mathrm{AT}(B_{N,\alpha}, r) \leq c_2 \cdot N^{1+\alpha} \log N.$$

  - *(large center) If $\alpha \in (0, 0.5)$ then there exist constants c_1, c_2 such that*

    $$c_1 \cdot N^{1+\alpha} \log N \leq \mathrm{AT}(B_{N,\alpha}, r) \leq c_2 \cdot N^{1+2\alpha} \log N.$$

Moreover, the fixation time $T(B_{N,\alpha}, r)$ satisfies the same inequalities.

As an immediate corollary, we obtain that for any fixed $r > 1$, both the absorption and the fixation time on a Star graph ($\alpha = 1$) are of the order of $\Theta(N^2 \log N)$. This is in alignment with earlier results [4, 5].

Third, we prove that under temperature initialization, analogous results can be achieved using Weighted bipartite graphs $W_{N,\alpha}$.

Theorem 3. *Fix $\alpha \in (0, 1]$ and $r > 1$. Let $W_{N,\alpha}$ be the Weighted bipartite graph. Then*

- $\mathrm{fp}(W_{N,\alpha}, r) \to 1 - 1/r^2$.
- *There exist constants c_1, c_2 such that*

  $$c_1 \cdot N^{1+\alpha} \log N \leq \mathrm{AT}(B_{N,\alpha}, r) \leq c_2 \cdot N^{1+\frac{3}{2}\alpha} \log N.$$

Moreover, the fixation time $T(B_{N,\alpha}, r)$ satisfies the same inequalities.

3 Formal proofs of theoretical results

3.1 Lower bound on absorption time

Here we show that for $r > 1$ no family of graphs with fixation probability bounded away from zero can have asymptotically smaller absorption time than the Complete graphs. Specifically, no amplifiers can absorb asymptotically faster than the Complete graphs. Recall that for the Complete graph on N vertices, both the fixation time and the absorption time is of the order of $\Theta(N \log N)$ (see Section 3.4).

Theorem 1. *Fix $r > 1$. Let G be any graph with $N \geq 2$ vertices and let $p = \mathrm{fp}(G, r)$ be the fixation probability of a single mutant under uniform initialization. Then*

$$\mathrm{AT}(G, r) \geq \frac{p}{r} \cdot N \cdot H_{N-1},$$

where $H_{N-1} = \frac{1}{1} + \frac{1}{2} + \cdots + \frac{1}{N-1} \geq \log N$. In particular, $\mathrm{AT}(G, r) \geq \frac{p}{r} \cdot N \log N$ for an arbitrary graph G and $\mathrm{AT}(A, r) \geq \frac{r-1}{r^2} \cdot N \log N$ for an arbitrary amplifier A.

Proof. Consider a modified Moran process M' that is identical with the standard Moran process, except that if the mutation goes extinct then in the next step we again initialize a single mutant uniformly at random and continue the process. Clearly, the modified process M' always terminates with the mutants fixating and its expected fixation time is given by $T'(G, r) = \frac{1}{p} \cdot \mathrm{AT}(G, r)$.

Given any subset X of the vertices, let p^X be the probability to gain a mutant in a single step from a configuration consisting of mutants at vertices of X and residents elsewhere. To gain a mutant, one of the $|X|$ mutants has to be selected for reproduction and then the offspring has to replace a resident. The probability of the first event alone equals $\frac{r|X|}{N+(r-1)|X|}$, hence we get an upper bound

$$p^X \leq \frac{r|X|}{N + (r-1)|X|} \leq \frac{r|X|}{N} \equiv p_{|X|}$$

that doesn't depend on X but only on $|X|$.

Finally, fix $k \in \{1, 2, \ldots, N-1\}$ and observe that any evolutionary trajectory in M' has to, at some point, reach a state with k mutants and gain another mutant from there. Hence, in expectation, the evolutionary trace spends at least $\frac{1}{p_k}$ steps in states corresponding to configurations with k mutants. By linearity of expectation, summing over k gives

$$T(G, r) = p \cdot T'(G, r) \geq p \sum_{k=1}^{N-1} \frac{N}{r \cdot k} = \frac{p}{r} \cdot N \cdot H_{N-1}$$

as desired. □

Remarks on the lower bound

Several remarks are in order.

First, we emphasize that the proof applies to all graphs, possibly containing directed edges, weighted edges, and/or self-loops.

Second, we note that the same proof goes through for any initialization scheme $\mathcal{S}$ (with $p = \mathrm{fp}(G, r)$ replaced by the fixation probability $p^{\mathcal{S}}$ under that initialization scheme $\mathcal{S}$). Specifically, it

applies to temperature initialization and also to schemes in which the first mutant is initialized to a fixed vertex.

Third, we discuss relationship between absorption time and fixation time. Note that Theorem 1 provides a lower bound on the absorption time $\mathrm{AT}(G,r)$ which is a weighted average of the fixation time $T(G,r)$ and the extinction time $\mathrm{ET}(G,r)$. Since the evolutionary trajectories leading to extinction are typically shorter than those leading to fixation, the fixation time tends to be even longer than the absorption time. In fact, the inequality $T(G,r) > \mathrm{AT}(G,r)$ holds for all undirected graphs G and all values $r > 1$ that we tested. On the other hand, there do exist directed graphs for which the opposite inequality $T(G,r) < \mathrm{AT}(G,r)$ holds. As an example, consider $r = 4$ and a graph G consisting of three vertices $\{u, v_1, v_2\}$ and edges $\{u \to v_1, u \to v_2, v_1 \leftrightarrow v_2\}$. Then we easily check that $T(G,4) = 3.25$ while $\mathrm{AT}(G,4) = 19.25$. In fact, in terms of fixation time, this graph G is even slightly faster than the complete graph K_3, as we have $T(K_3,4) = 3 + \frac{4}{7} > 3.25$.

3.2 α-Balanced bipartite graphs

In this subsection we analyze the α-Balanced bipartite graph $B_{N,\alpha}$. Recall that $B_{N,\alpha}$ consists of $c = N^{1-\alpha}$ vertices in the (smaller) center and N vertices in the outside part, each two vertices from different parts connected by an edge.

We prove the following theorem.

Theorem 2. *Fix $\alpha \in (0,1]$ and $r > 1$. Let $B_{N,\alpha}$ be the α-Balanced bipartite graph. Then*

- $\mathrm{fp}(B_{N,\alpha}, r) \to 1 - 1/r^2$.
  - *(small center) If $\alpha \in (0.5, 1)$ then there exist constants c_1, c_2 such that*
  $$c_1 \cdot N^{1+\alpha} \log N \le \mathrm{AT}(B_{N,\alpha}, r) \le c_2 \cdot N^{1+\alpha} \log N.$$
  - *(large center) If $\alpha \in (0, 0.5)$ then there exist constants c_1, c_2 such that*
  $$c_1 \cdot N^{1+\alpha} \log N \le \mathrm{AT}(B_{N,\alpha}, r) \le c_2 \cdot N^{1+2\alpha} \log N.$$

Moreover, the fixation time $T(B_{N,\alpha}, r)$ satisfies the same inequalities.

Martingales background

First, we recall the following facts about martingales (see [6]). Fix $r > 1$. Given a complete bipartite graph with v vertices at the outside part and c vertices in the center, the state (configuration) space can be parametrized by the number $0 \le i \le v$ of mutants in the outside part and the number $0 \le j \le c$ of mutants in the center. For each state (i,j), let $\mathrm{fp}(i,j)$ be the fixation probability starting from that state. There is a formula for $\mathrm{fp}(i,j)$ which can be computed as follows: Let

$$h_v = \frac{v + cr}{vr^2 + cr}, \qquad h_c = \frac{c + vr}{cr^2 + vr}$$

and for every state (i,j) define a potential function $\phi(i,j) = h_v^i \cdot h_c^j$. (Note that $\phi(i+1,j) = \phi(i,j) \cdot h_v$ and $\phi(i, j+1) = \phi(i,j) \cdot h_c$.) Then

$$\mathrm{fp}(i,j) = \frac{\phi(0,0) - \phi(i,j)}{\phi(0,0) - \phi(v,c)} = \frac{1 - \phi(i,j)}{1 - \phi(v,c)}.$$

For the rest of this section, we will be using these results for $c = N^{1-\alpha}$ and $v = N$.

Fixation probability

With the martingales background, the analysis of the fixation probability is relatively straightforward.

Lemma 1. Fix $\alpha \in (0,1]$ and $r > 1$. As $N \to \infty$, we have $\mathrm{fp}(B_{N,\alpha}, r) \to 1 - 1/r^2$.

Proof. The original mutant appears at the outside part with probability $N/(N+N^{1-\alpha}) \to 1$. Since $\phi(1,0) = h_v \to 1/r^2$ and $\phi(v,c) = h_v^v \cdot h_c^c < h_v^v \to 0$ as $N \to \infty$, we compute

$$\mathrm{fp}(B_{N,\alpha}, r) = \frac{1-\phi(1,0)}{1-\phi(v,c)} \to_{N\to\infty} 1 - 1/r^2.$$

□

Lower bound on fixation time

Next, we present the lower bounds for the absorption and fixation time. The idea is to consider the expected time t_k to gain one mutant in the outside part, if there are currently k mutants there. By bounding those times and summing up we obtain the following lemma.

Lemma 2. Fix $\alpha \in (0,1]$. Then

$$\mathrm{AT}(B_{N,\alpha}, r) = \Omega(N^{1+\alpha} \log N), \quad T(B_{N,\alpha}, r) = \Omega(N^{1+\alpha} \log N).$$

Proof. For the absorption time, we proceed as in the proof of Theorem 1, that is, we restart the process each time the mutants go extinct. The modified process M' always terminates with the mutants fixating and its expected fixation time is given by $T'(B_{N,\alpha}, r) = \frac{1}{\mathrm{fp}(B_{N,\alpha},r)} \cdot \mathrm{AT}(B_{N,\alpha}, r)$. Consider a state with $1 \le k \le N-1$ mutants in the outside part and $0 \le j \le c$ mutants at the center. Let $F = N + c + (r-1)(j+k) > N$ be the total fitness of the population. The probability that in the next step we gain one mutant in the outside part equals

$$\frac{r\cdot j}{F} \cdot \frac{N-k}{N} \le \frac{r \cdot c}{N^2} \cdot (N-k) \equiv p_k.$$

Since p_k is independent of j, the expected time to reach some state with $k+1$ mutants, starting in any state with k mutants in the outside part, is at least

$$\frac{1}{p_k} = \frac{1}{r} \cdot \frac{N^2}{c} \cdot \frac{1}{N-k} \equiv t_k.$$

In order to fixate, we need to pass through a state with k mutants in the outside part, for each $k = 1, \ldots, N-1$. By linearity of expectation,

$$\begin{aligned} \mathrm{AT}(B_{N,\alpha}, r) &= \mathrm{fp}(B_{N,\alpha}, r) \cdot T'(B_{N,\alpha}, r) \\ &\ge \mathrm{fp}(B_{N,\alpha}, r) \cdot \sum_{k=1}^{N-1} \frac{1}{r} \cdot \frac{N^2}{c} \cdot \frac{1}{N-k} \to \frac{r^2-1}{r^3} \cdot N^{1+\alpha} \cdot \sum_{k=1}^{N-1} \frac{1}{k} = \Theta(N^{1+\alpha} \log N). \end{aligned}$$

For the fixation time, we perform a standard construction to obtain a different modified process M'' that only includes the trajectories that lead to fixation. Specifically, we remove the state $(0,0)$

(the only state s with $\mathrm{fp}(s) = 0$) and, for any two other states s and t, we renormalize the transition probability $p(s \to t)$ to a new value $p''(s \to t) = p(s \to t) \cdot \frac{\mathrm{fp}(t)}{\mathrm{fp}(s)}$. It is a standard result that in this way we have constructed a Markov chain with only one absorbing state whose absorption time is equal to the fixation time of the original process, that is, $T(B_{N,\alpha}, r) = \mathrm{AT}''(B_{N,\alpha}, r)$. To get a lower bound for $\mathrm{AT}''(B_{N,\alpha}, r)$, we proceed as before.

Due to the renormalization, each p_k ($k = 1, \ldots, N-1$) gets multiplied by a ratio of two fixation probabilities that can be upper bounded by

$$\frac{\max_j\{\mathrm{fp}(k+1, j)\}}{\min_j\{\mathrm{fp}(k, j)\}}.$$

Note that for $k \geq 1$ the denominator is at least a constant (recall that $\mathrm{fp}(1, 0) \to 1 - 1/r^2$ for large N), hence the ratio can be further upper bounded by $1/c_0$ for any $c_0 < 1 - 1/r^2$ and $N \to \infty$. Hence $t''_k = 1/p''_k \geq c_0/p_k$. This gives

$$T(B_{N,\alpha}, r) = \mathrm{AT}''(B_{N,\alpha}, r) \geq \sum_{k=1}^{N-1} t''_k \geq c_0 \sum_{k=1}^{N-1} \frac{1}{r} \cdot \frac{N^2}{c} \cdot \frac{1}{N-k} \quad = \frac{c_0}{r} \cdot N^{1+\alpha} \cdot \sum_{k=1}^{N-1} \frac{1}{k} = \Theta(N^{1+\alpha} \log N)$$

as desired. □

Upper bound: "small" center

For the upper bound, we distinguish two cases. First, we assume that $\alpha \in (1/2, 1]$, that is $c = o(\sqrt{N})$.

The idea is to again work with the restarted process and moreover to split the set of states into *sections* as follows: section S_i consists of all the states with i mutants in the outside part. Then we consider a Markov chain $\mathcal{M}'$ whose nodes are the sections S_i. By construction, the only transitions with nonzero probability are of the form $S_i \to S_{i\pm1}$ or $S_i \to S_i$. In the following sequence of Lemmas, we provide upper bounds for the expected number of transitions from S_{i+1} to S_i and for the expected number of transitions within each S_i. Summing up, we obtain an upper bound for the fixation time in the original Markov chain.

Formally, fix i and let

- $f_{\max} = \max_j\{\mathrm{fp}(i, j)\}$ be the maximum fixation probability from a state in S_i. Clearly, $f_{\max}$ is attained in state (i, c).
- $g_{\min} = \min_j\{\mathrm{fp}(i+1, j)\}$ be the minimum fixation probability from a state in S_{i+1}. Clearly, $g_{\min}$ is attained in state $(i+1, 0)$.
- $q = \min_j\{q_j\}$ where q_j is the probability that an evolutionary trajectory starting at $(i+1, j)$ fixates at (v, c) before visiting any state in S_i.

First, since $\alpha > 1/2$ we have the following:

Lemma 3. $h^c_c \to_{N\to\infty} 1$ and $f_{\max} < g_{\min}$ (for large enough N)

Proof. We have

$$h_c^c \approx \left(1 - \frac{r - 1/r}{N^\alpha}\right)^{(N^{1-\alpha})}$$

For $N \to \infty$ we have $N^\alpha \to \infty$. If the parenthesis was raised to power N^α, the limit would have been $\exp(-(r - 1/r))$, a constant. Since $N^{1-\alpha} = o(N^\alpha)$ for $\alpha > 1/2$, we have $\lim_{N\to\infty} h_c^c = 1$. Hence $h_c^c > h_v$, then $\phi(i, c) = h_c^c \cdot \phi(i, 0) > h_v \cdot \phi(i, 0) = \phi(i + 1, 0)$ and thus $f_{\max} = \mathrm{fp}(i, c) < \mathrm{fp}(i + 1, 0) = g_{\min}$ as desired. □

We aim to bound q from below and use it to bound the expected number X of transitions from (any state in) S_{i+1} to (any state in) S_i from above.

Lemma 4. $q \geq \frac{g_{\min} - f_{\max}}{1 - f_{\max}}$

Proof. Let's run an evolutionary trajectory from some state $(i+1, j)$ in S_{i+1}. The trajectory can't go extinct without hitting S_i. Conditioning on if the trajectory first fixates or hits S_i, we can write

$$g_{\min} \leq \mathrm{fp}(i + 1, j) \leq q_j \cdot 1 + (1 - q_j) \cdot f_{\max}$$

which rewrites as

$$q_j \geq \frac{g_{\min} - f_{\max}}{1 - f_{\max}}.$$

This is true for every j, hence it is true for $q = \min_j\{q_j\}$ too. □

Let X be a random variable counting the transitions from any state in S_{i+1} to any state in S_i, starting from any state.

Lemma 5. $\mathbb{E}[X] \leq \frac{1-q}{q} = \frac{1 - g_{\min}}{g_{\min} - f_{\max}}$.

Proof. Any two transitions from section S_{i+1} to section S_i are necessarily separated by an intermediate visit to section S_{i+1}. Any time we are in section S_{i+1}, with probability at least q we fixate before hitting section S_i again. Hence

$$E[X] \leq q \cdot 0 + (1 - q)(1 + E[X]).$$

Rewriting and using the bound for q we obtain

$$\mathbb{E}[X] \leq \frac{1 - q}{q} = \frac{1}{q} - 1 = \frac{1 - f_{\max}}{g_{\min} - f_{\max}} - 1 = \frac{1 - g_{\min}}{g_{\min} - f_{\max}}.$$

□

Rewriting $g_{\min}$ and $f_{\max}$ in terms of h_v, h_c we deduce that $\mathbb{E}[X]$ is constant.

Lemma 6. $\mathbb{E}[X] \leq \frac{1}{r^2 - 1}$ (for large enough N)

Proof. Recall that $\mathrm{fp}(i, j) = \frac{1-\phi(i,j)}{d}$ where $d = 1 - \phi(v, c)$ doesn't depend on i, j. Plugging this in the bound from Lemma 5 we get

$$\mathbb{E}[X] \leq \frac{1 - g_{\min}}{g_{\min} - f_{\max}} = \frac{1 - \frac{1-\phi(i+1,0)}{d}}{\frac{1-\phi(i+1,0)}{d} - \frac{1-\phi(i,c)}{d}}$$

$$= \frac{d - (1 - \phi(i + 1, 0))}{1 - \phi(i + 1, 0) - (1 - \phi(i, c))} < \frac{\phi(i + 1, 0)}{\phi(i, c) - \phi(i + 1, 0)}.$$

Using the definition $\phi(i,j) = h_v^i h_c^j$ and dividing by h_v^i this can be further rewritten as

$$\mathbb{E}[X] < \frac{\phi(i+1,0)}{\phi(i,c) - \phi(i+1,0)} = \frac{h_v}{h_c^c - h_v} \to_{N\to\infty} \frac{1/r^2}{1 - 1/r^2}$$

as desired. $\square$

Let $\mathbb{E}[\mathcal{L}_i]$ be the expected number of "looping" transitions of the form $S_i \to S_i$ before a transition of the form $S_i \to S_{i\pm 1}$ occurs (or the process reaches an absorbing state). The following lemma bounds $\mathbb{E}[\mathcal{L}_i]$ from above.

Lemma 7. For $i = 1, 2, \ldots, N-1$ we have $\mathbb{E}[\mathcal{L}_i] \le \frac{r \cdot N(N+c)}{c \cdot \min\{i, N-i\}} - 1$. Moreover, $\mathbb{E}[\mathcal{L}_0] \le r(N+c) - 1$ and $\mathbb{E}[\mathcal{L}_N] \le r(N+c) - 1$.

Proof. Crudely (not caring about r). First, let $i = 1, \ldots, N-1$. We pick a vertex in the center with probability at least $\frac{1 \cdot c}{r(N+c)}$. No matter its type, there are at least $\min\{i, N-i\}$ vertices of the other type at the outside part. Hence with probability

$$p \ge \frac{1 \cdot c}{r(N+c)} \cdot \frac{\min\{i, N-i\}}{N}$$

we transition to section $S_{i\pm 1}$ in one step. As before, we get the result from $\mathbb{E}[\mathcal{L}_i] \le \frac{1}{p} - 1$. Second, if $i = 0$ or $i = N$ and we are not in an absorbing state then there exists a vertex in the center whose type is different to the type of all vertices in the outside part. Hence $p \ge \frac{1}{r(N+c)}$ and we conclude as in the first case. $\square$

We are ready to sum those contributions up.

Lemma 8. If $\alpha \in (1/2, 1]$, $c = N^{1-\alpha} = o(\sqrt{N})$ and $r > 1$ then

$$\mathrm{AT}(B_{N,\alpha}, r) = O\left(\frac{N^2}{c} \cdot \log N\right) = O(N^{1+\alpha} \log N).$$

Proof. As in the proof of Theorem 1 we restart the process anytime the mutants fixate. Consider the one-dimensional Markov chain $\mathcal{M}'$ whose vertices are the sections S_i, $i = 0, \ldots, N$. Fix $i \in \{1, \ldots, N-1\}$ and let $f(r) = \frac{1}{r^2 - 1}$. On average, there are at most $f(r)$ transitions $S_{i+1} \to S_i$. Also, on average there are at most $f(r)$ transitions $S_i \to S_{i-1}$, hence there are at most $f(r)+1$ transitions $S_{i-1} \to S_i$ for a total of at most $2f(r)+1$ transitions from outside of S_i to S_i. Similarly, on average there are at most $f(r)$ transitions into S_0 and at most $f(r)+1$ transitions into S_N. Every time there is a transition into S_i, there are on average $\mathbb{E}[\mathcal{L}_i]$ transitions within S_i. By linearity of expectation, the total expected number of transitions is at most

$$\begin{aligned}
\mathrm{AT}(B_{N,\alpha}, r) &= \mathrm{fp}(B_{N,\alpha}, r) \cdot T'(B_{N,\alpha}, r) \\
&\le \mathrm{fp}(B_{N,\alpha}, r) \cdot \left(f(r) \cdot r(N+c) + \left(\sum_{i=1}^{N-1} (2f(r)+1)(1 + \mathbb{E}[\mathcal{L}_i]) \right) + (f(r)+1) \cdot r(N+c) \right) \\
&= \mathrm{fp}(B_{N,\alpha}, r) \cdot (2f(r)+1) \cdot r(N+c) \cdot \left(1 + \frac{N}{c} \sum_{i=1}^{N-1} \frac{1}{\min\{i, N-i\}} \right) = \Theta\left(\frac{N^2}{c} \cdot \log N\right),
\end{aligned}$$

where the last equality follows from the sum being $\Theta(2\log(N/2)) = \Theta(\log N)$ and from $c = o(N)$. $\square$

Upper bound: "large" center

Note that the argument used for small center fails for $\alpha \leq 1/2$ because the difference $g_{\min} - f_{\max}$ becomes zero or even negative. Indeed, for $\alpha = 1/2$ we have $h_c^c \to_{N\to\infty} \exp(-(r - 1/r)) < 1/r^2$ and for $\alpha < 1/2$ the inequality is even stricter. However an analogous argument can be made to work if we split the state space into different "tilted" sections, taking α into account. The idea of the proof is that we fix $\alpha \in (0, 1/2)$, consider large N, and look at a complete bipartite graph $B_{N,\alpha}$. We assume that r is such that there exists an integer t called "tilt" satisfying $h_c^t = h_v$. This assumption guarantees that the states $(i, j + t)$ and $(i + 1, j)$ are assigned exactly the same potential. We can then split the state (configuration) space into $\Theta(N)$ sections where each section is not a vertical line but a set of c states that looks like a line tilted with slope $-t$ (see figure). We then proceed as before, providing an upper bound for the number of transitions across sections and within sections. The result follows by summing up.

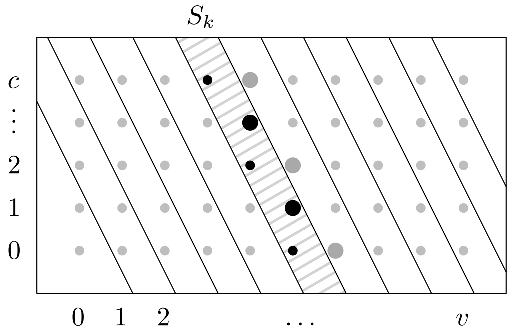

Figure 4: **Tilted sections of $B_{N,\alpha}$.** For $\alpha \in (0, 1/2)$ we split the state (configuration) space into $\Theta(N)$ "tilted" sections S_k. Here the tilt is $t = 2$. The maximum potential within S_k is attained at any thick black vertex, the minimum potential within S_{k+1} is attained at any thick grey vertex.

In the rest of the section we formalize this idea. First, we define the (tilted) sections. Let $s = v + \lfloor c/t \rfloor$ and for $k = 0, \ldots, s$ let

$$S_k = \{(i, j) : i + \lfloor j/t \rfloor = k\} .$$

As before, we fix k and define

- $f_{\max} = \max\{\text{fp}(i, j) : (i, j) \in S_k\}$,
- $g_{\min} = \min\{\text{fp}(i, j) : (i, j) \in S_{k+1}\}$, and
- $q = \min_{(i,j)\in S_{k+1}}\{q_{(i,j)}\}$ where $q_{(i,j)}$ is the probability that an evolutionary trajectory starting at state (i, j) belonging to S_{k+1} fixates at (v, c) before visiting any state in S_k.

Clearly, $f_{\max}$ is attained for any "top" state of S_k within its column (possibly not in the "top" row). Similarly, $g_{\min}$ is attained in state "bottom" state of S_{k+1} (possibly not in the "bottom" row). Note that by construction, those two states are assigned potentials that differ by a factor of h_c.

As before, let X be a random variable counting the transitions from any state in S_{k+1} to any state in S_k, starting from any state. The following lemma bounds $\mathbb{E}[X]$ from above. Note that this time the bound is super-constant.

Lemma 9. $\mathbb{E}[X] \leq \frac{r}{r^2-1} \cdot N^\alpha$ (for large N)

Proof. Note that Lemma 4 and Lemma 5 are valid for tilted sections too. Let (i,j) be some state in S_k for which the value $f_{\max}$ is attained. Then $g_{\min}$ is attained at a state whose potential is equal to $h_c \cdot \phi(i,j)$. We continue as in the proof of Lemma 6 to get

$$\mathbb{E}[X] \leq \frac{1-g_{\min}}{g_{\min}-f_{\max}} = \frac{h_c \cdot \phi(i,j) - \phi(v,c)}{\phi(i,j) - h_c \cdot \phi(i,j)} < \frac{h_c}{1-h_c}.$$

Since for large N we have $h_c \approx 1-(1-1/r)/N^\alpha$, the right-hand side can be approximated as

$$\frac{h_c}{1-h_c} < \frac{1}{1-h_c} = \frac{N^\alpha}{r-1/r} = \frac{r}{r^2-1} \cdot N^\alpha.$$

□

It remains to bound the expected number $\mathbb{E}[\mathcal{L}_k]$ of the "looping" transitions of the form $S_k \to S_k$ before a transition of the form $S_k \to S_{k\pm1}$ occurs. This is done as before, observing that any two states that differ only in the number of mutants in the outside part of the graph always lie in different sections. Hence Lemma 7 holds.

Finally, we prove the last inequality in Theorem 2.

Lemma 10. If $\alpha \in (0, 1/2)$ then

$$\mathrm{AT}(B_{N,\alpha}, r) = O(N^{1+2\alpha} \log N).$$

Proof. As before, let $f(r) = \frac{r}{r^2-1} \cdot N^\alpha$. By linearity of expectation, the total expected number of transitions is

$$\begin{aligned}\mathrm{AT}(B_{N,\alpha}, r) &\leq \mathrm{fp}(B_{N,\alpha}, r) \cdot \left(f(r) \cdot r(N+c) + \left(\sum_{i=1}^{N-1} (2f(r)+1)(1+\mathbb{E}[\mathcal{L}_i]) \right) + (f(r)+1) \cdot r(N+c) \right) \\ &= \mathrm{fp}(B_{N,\alpha}, r) \cdot (2f(r)+1) \cdot r(N+c) \cdot \left(1 + \frac{N}{c} \sum_{i=1}^{N-1} \frac{1}{\min\{i, N-i\}} \right) = O\left(N^\alpha \cdot N \cdot \frac{N}{c} \cdot \log N \right) \\ &= O(N^{1+2\alpha} \log N)\end{aligned}$$

and the result follows. □

Finally, we observe that an upper bound on $\mathrm{AT}(B_{N,\alpha}, r)$ immediately implies an asymptotically matching upper bound on $T(B_{N,\alpha}, r)$.

Lemma 11. Fix $r > 1$ and $\alpha > 0$. If $\mathrm{AT}(B_{N,\alpha}, r) = O(N^\alpha \log N)$ then $T(B_{N,\alpha}, r) = O(N^\alpha \log N)$

Proof. Since the absorption time is a weighted average of the fixation time and the extinction time, we have

$$\mathrm{AT}(B_{N,\alpha}, r) = \mathrm{fp}(B_{N,\alpha}, r) \cdot T(B_{N,\alpha}, r) + (1 - \mathrm{fp}(B_{N,\alpha}, r)) \cdot \mathrm{ET}(B_{N,\alpha}, r)) \geq \mathrm{fp}(B_{N,\alpha}, r) \cdot T(B_{N,\alpha}, r)$$

Since $\mathrm{fp}(B_{N,\alpha}, r) \to 1 - 1/r^2$ as $N \to \infty$ and $\mathrm{AT}(B_{N,\alpha}, r) = O(N^\alpha \log N)$, the result follows. □

Altogether, Lemmas 1, 2, 8, 10 and 11 prove all the statements of Theorem 2.

3.3 Weighted bipartite graphs

In this section we analyze the Weighted bipartite graphs $W_{N,\alpha}$ under temperature initialization. Recall that $W_{N,\alpha}$ is a complete bipartite graph with one (smaller) part of size $c = N^{1-\alpha}$, one (larger) part of size N, and every vertex of the larger part having a self-loop of such a weight w that $N^{-\alpha/2} = \frac{N^{1-\alpha}}{w+N^{1-\alpha}}$.

We prove the following theorem.

Theorem 3. *Fix $\alpha \in (0,1)$ and $r > 1$. Let $W_{N,\alpha}$ be the Weighted bipartite graph. Then*

- $\text{fp}(W_{N,\alpha}, r) \to 1 - 1/r^2$.
- *There exist constants c_1, c_2 such that*

$$c_1 \cdot N^{1+\alpha} \log N \le \text{AT}(B_{N,\alpha}, r) \le c_2 \cdot N^{1+\frac{3}{2}\alpha} \log N.$$

Moreover, the fixation time $T(B_{N,\alpha}, r)$ satisfies the same inequalities.

Martingales for Weighted bipartite graphs

First, we recall more martingales background.

Fix $r > 1$. Given integers v, c, and a real number $q \in (0,1)$, let $W(c, v, q)$ be a Weighted complete bipartite graph with c vertices in the smaller part (center) and v vertices at the larger (outside) part, each of them with an extra self-loop of such weight w that $q = \frac{c}{w+c}$ is the probability that when a vertex in the larger part is selected for reproduction, its offspring replaces one of the vertices in the smaller part (as opposed to replacing its parent via the self-loop). Then, as with the unweighted complete bipartite graphs, the state space can be parametrized by the number $0 \le i \le v$ of mutants in the outside part and the number $0 \le j \le c$ of mutants in the center and the fixation probabilities from all the states can be computed similarly to above, with v replaced by $v \cdot q$.

Namely, let

$$h_v = \frac{qv + cr}{qvr^2 + cr}, \qquad h_c = \frac{c + qvr}{cr^2 + qvr}$$

and for every state (i, j) of i mutants in the outside part and j mutants in the center, define a potential function $\phi(i, j) = h_v^i \cdot h_c^j$. Then we easily check the fixation probability from a state (i, j) is given by

$$\text{fp}(i, j) = \frac{\phi(0, 0) - \phi(i, j)}{\phi(0, 0) - \phi(v, c)} = \frac{1 - \phi(i, j)}{1 - \phi(v, c)}.$$

Fixation probability

With the extra martingales background, the analysis of the fixation probability is again relatively straightforward.

Lemma 12. $\text{fp}_{\mathcal{T}}(W_{N,\alpha}, r) \to 1 - 1/r^2$.

Proof. The first mutant is introduced in the center with probability proportional to $q \cdot v$ and to the outside part with probability proportional to $c + (1-q)v$. Since $q = o(1)$, it is introduced to the outside part with high probability. The fixation probability $\mathrm{fp}(1,0)$ starting from a state with a single mutant in the outside part satisfies

$$\mathrm{fp}(1,0) = \frac{1-\phi(1,0)}{1-\phi(v,c)} = \frac{1-h_v}{1-h_v^v h_c^c}.$$

Since $h_v \approx \frac{1}{r^2}$ and $h_v^v \to 0$ as $N \to \infty$, we have $\mathrm{fp}(1,0) \to 1 - 1/r^2$. □

Fixation time

The arguments for fixation time are direct translations of arguments for (unweighted) α-Balanced bipartite graphs (see Section 3.2). For the lower bound, Lemma 2 still applies. For the upper bound, we proceed analogously.

Lemma 13. If $\alpha \in (0,1)$ and $r > 1$ then $T_{\mathcal{T}}(W_{N,\alpha}, r) = O(N^{1+\frac{3}{2}\alpha} \log N)$.

Proof. Fixing k and considering the section S_k, we denote by X the expected number of transitions from any state in S_{k+1} to any state in S_k. As in Lemmas 6 and 9 we get

$$\mathbb{E}[X] \le \frac{h_c}{1-h_c} = \Theta(N^{\alpha/2}).$$

Lemma 7 then yields

$$T_{\mathcal{T}}(W_{N,\alpha}) = O\left(N^{\alpha/2} \cdot N \cdot \frac{N}{c} \cdot \log N\right) = O(N^{1+\frac{3}{2}\alpha} \log N).$$

□

Altogether, Lemmas 2, 11, 12 and 13 prove all the statements of Theorem 3

3.4 Time on Complete graph

For reference purposes we compute the absorption, fixation, and extinction times of a single advantageous mutant ($r > 1$) on a Complete graph, using the standard difference method.

Theorem 4. *Fix $r > 1$ and let K_N be the Complete graph on N vertices. Then*

$$\begin{aligned}
\mathrm{AT}(K_N, r) &= (N-1)H_{N-1} \cdot \frac{r+1}{r} + (N-1) \cdot \log(1-1/r) - \frac{1}{r(r-1)} + o(1), \\
T(K_N, r) &= (N-1)H_{N-1} \cdot \frac{r+1}{r-1} + (N-1) \cdot \frac{r+1}{r-1} \log(1-1/r) + o(N), \\
\mathrm{ET}(K_N, r) &= (N-1) \cdot \log\left(\frac{r}{r-1}\right) + o(N).
\end{aligned}$$

In particular for $r = 1+s$, $s > 0$ small, we have $\mathrm{AT}(K_N, r) \approx 2 \cdot N \log N$, $T(K_N, r) \approx \frac{2}{s} \cdot N \log N$, *and* $\mathrm{ET}(K_N, r) \approx \frac{1}{s} \cdot N$.

Proof. First, we compute the absorption time, then the fixation time and finally the extinction time.

Absorption time. Fix N and r and for $k = 0, \dots, N$ let T_k be the expected absorption time from a state with k mutants. Clearly $T_0 = T_N = 0$ and for $k = 1, \dots, N-1$ we have

$$T_k = 1 + p(k,k)T_k + p(k,k-1)T_{k-1} + p(k,k+1)T_{k+1},$$

where $p(i,j)$ is the transition probability from a state with i mutants to a state with j mutants. Specifically, we have

$$p(k,k-1) = \frac{N-k}{N+(r-1)k} \cdot \frac{k}{N-1}. \qquad \text{and} \qquad p(k,k+1) = \frac{r \cdot k}{N+(r-1)k} \cdot \frac{N-k}{N-1}.$$

Plugging in those values of $p(i,j)$, the above equation can be rewritten as

$$T_{k+1} - T_k = \frac{1}{r}\left(T_k - T_{k-1}\right) - \left(\frac{N-1}{rk} + \frac{N-1}{N-k}\right).$$

Setting $\Delta_k \equiv T_k - T_{k-1}$ and $x_k = \frac{N-1}{rk} + \frac{N-1}{N-k}$ this further rewrites as

$$\Delta_{k+1} = \frac{1}{r}\Delta_k - x_k.$$

Specifically, we have $\Delta_1 = T_1 - T_0 = T_1$ and $\Delta_1 + \cdots + \Delta_N = T_N - T_0 = 0$. Let's write

$$\begin{aligned}
\Delta_2 &= \frac{1}{r}\Delta_1 - x_1, && (1)\\
\Delta_3 &= \frac{1}{r}\Delta_2 - x_2, && (2)\\
\Delta_4 &= \frac{1}{r}\Delta_3 - x_3, && (3)\\
&\cdots\\
\Delta_N &= \frac{1}{r}\Delta_{N-1} - x_{N-1}, && (N-1)
\end{aligned}$$

We aim to express each Δ_k in terms of Δ_1 only. Summing up $\frac{1}{r}(1) + (2)$ gives

$$\Delta_3 = \frac{1}{r^2}\Delta_1 - \left(x_2 + \frac{1}{r}x_1\right)$$

Similarly, summing up $\frac{1}{r^2}(1) + \frac{1}{r}(2) + (3)$ gives

$$\Delta_4 = \frac{1}{r^3}\Delta_1 - \left(x_3 + \frac{1}{r}x_2 + \frac{1}{r^2}x_1\right)$$

and similarly all the way up to

$$\Delta_N = \frac{1}{r^{N-1}}\Delta_1 - \left(x_{N-1} + \frac{1}{r}x_{N-2} + \cdots + \frac{1}{r^{N-2}}x_1\right).$$

Summing up all of them, together with an extra equation $\Delta_1 = \Delta_1$, we get

$$0 = \Delta_1 + \cdots + \Delta_N = \Delta_1 \left(1 + \cdots + \frac{1}{r^{N-1}}\right) - \big(x_1(1+\cdots+1/r^{N-2}) + x_2(1+\cdots+1/r^{N-3}) + \cdots + x_{N-1}\cdot 1\big)$$

and in turn

$$\Delta_1 = \frac{1-1/r}{1-1/r^N} \cdot \frac{x_1(1-1/r^{N-1}) + x_2(1-1/r^{N-2}) + \cdots + x_{N-1}(1-1/r)}{1-1/r}$$
$$= \frac{1}{1-1/r^N}\Big(\underbrace{\sum_{k=1}^{N-1} x_k}_{A} - \underbrace{\sum_{k=1}^{N-1} \frac{x_k}{r^{N-k}}}_{B}\Big).$$

For A we easily get $A = \frac{r+1}{r}(N-1)H_{N-1}$. For B, we plug in $x_k = \frac{N-1}{rk} + \frac{N-1}{N-k}$, split $B = B_1 + B_2$ and separately compute the sums using a well-known limit

$$B_2 = (N-1)\sum_{i=1}^{N-1} \frac{1}{N-k} \cdot \frac{1}{r^{N-k}} = (N-1)\big(\log(1-1/r) + O(1/r^N)\big) \to (N-1)\log(1-1/r) + o(1)$$

and an approximation

$$B_1 = \frac{1}{r}\sum_{k=1}^{N-1} \frac{N-1}{N-k}\frac{1}{r^k} = \frac{1}{r}\sum_{k=1}^{N-1} \frac{1}{r^k} + E(N) = \frac{1}{r(r-1)} + o(1)$$

whose error term

$$E(N) = \sum_{k=1}^{N-1} \frac{k-1}{N-k} \cdot \frac{1}{r^k}$$

tends to 0, because the sum S_1 over the first $\sqrt[3]{N}$ terms satisfies

$$S_1 \le \sqrt[3]{N} \cdot \frac{\sqrt[3]{N}}{N - \sqrt[3]{N}} \cdot \frac{1}{r} < \frac{1}{\sqrt[3]{N}} \cdot \frac{1}{r} \to 0$$

and the sum S_2 of the remaining terms satisfies

$$S_2 \le (N - \sqrt[3]{N}) \cdot \frac{N}{1} \cdot \frac{1}{r^{\sqrt[3]{N}}} \to 0.$$

This concludes the proof of the absorption time.

Fixation time. We proceed similarly.

As before, we fix N and r and for $k = 1, \ldots, N$ we let T_k be the expected (conditional) fixation time from a state with k mutants (for $k = 0$ we define $T_0 = 0$). Then $T_N = 0$ and for $k = 1, \ldots, N-1$ we have

$$\mathrm{fp}_k T_k = \mathrm{fp}_k + p(k,k) \cdot \mathrm{fp}_k T_k + p(k,k-1) \cdot \mathrm{fp}_{k-1} T_{k-1} + p(k,k+1) \cdot \mathrm{fp}_{k+1} T_{k+1},$$

where $\text{fp}_i = \frac{1-1/r^i}{1-1/r^N}$ are the fixation probabilities and $p(i,j)$ are the transition probabilities. Setting $\Delta_k \equiv \text{fp}_k\, T_k - \text{fp}_{k-1}\, T_{k-1}$ and $x_k \equiv \frac{\text{fp}_k}{p(k,k+1)}$ this can be rewritten as

$$\Delta_{k+1} = \frac{1}{r}\Delta_k - x_k.$$

Specifically, we have $\Delta_1 = \text{fp}_1 \cdot T_1 - \text{fp}_0 \cdot T_0 = \text{fp}_1 \cdot T_1$ and $\Delta_1 + \cdots + \Delta_N = \text{fp}_N \cdot T_N - \text{fp}_0 \cdot T_0 = 0 - 0 = 0$. As before, we obtain

$$(1-1/r^N)\cdot\Delta_1 = \underbrace{\sum_{k=1}^{N-1} x_k}_{A} - \underbrace{\sum_{k=1}^{N-1} \frac{x_k}{r^{N-k}}}_{B}.$$

This time, $p(k,k+1) = \frac{rk}{N+(r-1)k}\cdot\frac{N-k}{N-1}$ and thus

$$x_k = \frac{\text{fp}_k}{p(k,k+1)} = \frac{1-1/r^k}{1-1/r^N}\cdot\left(\frac{r-1}{r} + \frac{N}{rk}\right)\cdot\frac{N-1}{N-k}$$

and

$$A = \sum_{k=1}^{N-1} x_k = \frac{N-1}{1-1/r^N}\cdot\sum_{k=1}^{N-1}(1-1/r^k)\cdot\left(\frac{1}{N-k}+\frac{1}{rk}\right).$$

Multiplying out the two parentheses we get

$$X \equiv \sum_{k=1}^{N-1}\frac{1}{N-k}+\frac{1}{rk} = (1+1/r)\cdot H_{N-1}$$

and

$$Y \equiv \sum_{k=1}^{N-1}\frac{1}{r^k(N-k)} \to 0, \quad Z \equiv \sum_{k=1}^{N-1}\frac{1}{r^k\cdot rk} \to \frac{1}{r}\log(1-1/r).$$

Hence

$$A = \frac{N-1}{1-1/r^N}\cdot(X+Y+Z) = (1+1/r)\cdot(N-1)H_{N-1} + \frac{1}{r}\log(1-1/r)\cdot(N-1) + o(N).$$

We proceed with B analogously. This time, the only combination that survives is

$$\sum_{k=1}^{N-1}\frac{1}{r^{N-k}}\cdot\frac{1}{N-k} \to \log(1-1/r),$$

hence $B = \log(1-1/r)\cdot(N-1)+o(N)$.

In total, we get

$$(1-1/r^N)\cdot\frac{1-1/r}{1-1/r^N}\cdot T_1 = (1-1/r^N)\cdot\Delta_1 = A+B = \frac{r+1}{r}\cdot(N-1)H_{N-1} + \frac{r+1}{r}\log(1-1/r)\cdot(N-1)+o(N)$$

and finally the desired

$$T(N,r) = T_1 = \frac{r+1}{r-1}\cdot(N-1)H_{N-1} + \frac{r+1}{r-1}\log(1-1/r)\cdot(N-1)+o(N).$$

Extinction time. A formula for the extinction time follows easily from the absorption time and the fixation time.

It suffices to note that $\mathrm{AT}_1 = \mathrm{fp}_1 \cdot T_1 + (1 - \mathrm{fp}_1) \cdot \mathrm{ET}_1$ and plug in the expressions for AT_1 and T_1. The $N \log N$ term cancels out and we are left with

$$\mathrm{ET}_1 = -\log(1 - 1/r) \cdot (N - 1) + o(N).$$

□

4 Details of figure legends

Here we present details of figures from the main text.

Figures 2, 3. **a, b** All the values are computed by numerically solving large systems of linear equations (see e.g. [7]). **d,** The values for the Complete graph, Star, Cycle, and all the 50 complete bipartite graphs of size 100 are computed numerically as above, the values for the other graphs are approximations obtained by simulating the Moran process 10^5 times and taking the average. The other graphs shown are randomly generated trees (100×), randomly generated sparse Erdős–Rényi graphs (100×, $p = 0.03$), Stars with additional 10, 30, 50, 100 random edges (10× each), and Cycles with additional 1, 3, 5, 10 random edges (5× each). In Figure 3d, we moreover show, for each $k \in \{1, 2, \ldots, 40\}$, a complete bipartite graph $K_{k,100-k}$ with weighted self-loops at each vertex of the larger part that maximize the fixation probability.

Figure 4. As above, all the values plotted are computed by numerically solving large systems of linear equations and using the formula for the effective rate of evolution. In **a, c**, we fix $N = 100$, $r = 1.1$, and vary $\mu \in \{x^0, \ldots, x^{45}\}$ where $x = \sqrt[5]{2}$ (that is, $\mu \in [1, 10^{-9}]$). In **b, d**, we fix $r = 1.1$ and $N\mu \in \{10^{-2}, 10^{-3}, 10^{-4}\}$ and vary $N \in \{10, 20, \ldots, 500\}$.

5 Future research directions

Here we list several interesting theoretical questions regarding the tradeoff between fixation probability and fixation time. Recall that Theorem 1 implies that no amplifiers are asymptotically faster (in terms of absorption time) than the Complete graphs of the same size. However, in principle, there could still exist amplifiers faster by a constant factor (in terms of either absorption or fixation time). On the other hand, we presented a simple directed graph that achieves slightly shorter fixation time than the Complete graph of the same size (for specific value of r).

1. Can the Complete graph be beat in both probability and time? In other words, does there exist N, $r > 1$ and a graph G on N vertices such that $T(G, r) < T(K_N, r)$ and $\mathrm{fp}(G, r) > \mathrm{fp}(K_N, r)$?

2. Do there exist graphs that are faster than the Complete graph by a constant factor? In other words, do there exist $c < 1$ and $r > 1$ and a family G_N of graphs (possibly suppressors) such that $\mathrm{AT}(G_N, r) < c \cdot \mathrm{AT}(K_N, r)$? What if we insist that the graphs G_N also amplify the fixation probability?

3. Do there exist graphs that are asymptotically faster than the Complete graphs? In other words, do there exist $r > 1$ and a family G_N of graphs such that $\mathrm{AT}(G_N, r) = o(\mathrm{AT}(K_N, r))$? By Theorem 1, such graphs would have to be strong suppressors of selection.

Moreover, how do the answers change if we insist that the graphs are undirected and/or that the results hold for all $r > 1$ as opposed to a single specific $r > 1$?

References

[1] Cormen TH. Introduction to algorithms. MIT press; 2009.

[2] Díaz J, Goldberg LA, Richerby D, Serna M. Absorption time of the Moran process. Random Structures & Algorithms. 2016;49(1):137–159.

[3] Adlam B, Chatterjee K, Nowak M. Amplifiers of selection. In: Proc. R. Soc. A. vol. 471; 2015. p. 20150114.

[4] Hadjichrysanthou C, Broom M, Rychtář J. Evolutionary games on star graphs under various updating rules. Dynamic Games and Applications. 2011;1(3):386.

[5] Askari M, Samani KA. Analytical calculation of average fixation time in evolutionary graphs. Physical Review E. 2015;92(4):042707.

[6] Monk T, Green P, Paulin M. Martingales and fixation probabilities of evolutionary graphs. Proc R Soc A Math Phys Eng Sci. 2014;470(2165):20130730.

[7] Hindersin L, Möller M, Traulsen A, Bauer B. Exact numerical calculation of fixation probability and time on graphs. Biosystems. 2016;150:87–91.
